# A Phenomenological Coupled Model for Ion Transport and Deformation in Superabsorbent Polymers in Calcium-Containing Solutions

**DOI:** 10.3390/gels12070606

**Published:** 2026-07-07

**Authors:** Qing Jiang, Yu Fu, Qijun Yu

**Affiliations:** 1 College of Civil Engineering, Hefei University of Technology, Hefei 230009, China; 2 Engineering Research Center of Low-Carbon Technology and Equipment for Cement-Based Materials, Ministry of Education, Hefei 230009, China; 3 Anhui Key Laboratory of Civil Engineering Structures and Materials, Hefei University of Technology, Hefei 230009, China; 4 School of Materials Science and Engineering, South China University of Technology, Guangzhou 510641, China

**Keywords:** superabsorbent polymer, ion transport, deformation, coupling model, calcium ion, numerical simulation

## Abstract

Understanding the absorption and desorption behavior of superabsorbent polymers (SAPs) in ionic environments is critical for their practical applications. Ion exchange between monovalent counterions within the SAP and multivalent cations (e.g., Ca^2+^) in solution not only induces macroscopic desorption but also generates non-uniform internal strain, creating a complex feedback loop with ion transport. This study establishes a phenomenological coupled model that integrates Fickian diffusion for ion transport with an elastic wave equation for SAP deformation. The coupling is realized through deformation-dependent diffusion coefficients and an ion-concentration-modulated elastic modulus, with the latter described by a first-order linear relationship over a limited range. Taking Ca^2+^ as a representative divalent cation, we systematically investigate the effects of solution concentration, SAP particle size, and ion dissociation degree. The model predicts several non-intuitive phenomena, including transient internal free Ca^2+^ concentrations exceeding the boundary concentration by up to ~15% and concentration gradient inversions for small SAP particles (radius 75 μm) at later times. Characteristic absorption time constants τ_a_ range from 98 s to 179 s depending on particle size and Ca^2+^ level. Simulated total Ca^2+^ uptake agrees with experimental data within an 8% mean relative error. The model is validated against macroscopic absorption/desorption curves and total Ca^2+^ uptake, while the predicted internal concentration and strain fields remain to be confirmed by spatially resolved experiments. These findings provide new mechanistic insights into the chemo-mechanical coupling in SAPs and offer guidance for their tailored design.

## 1. Introduction

Superabsorbent polymers (SAPs), a class of lightly cross-linked hydrogels, possess an exceptional capacity to absorb and retain aqueous solutions up to hundreds of times their own mass. This remarkable property underpins their critical roles in a diverse array of fields, including agriculture (for water retention) [1], hygiene products [2], drug delivery systems [3], and (as internal curing agents in) construction materials [4].

The performance of SAPs in these applications is fundamentally governed by their swelling and deswelling kinetics. While experimental investigations are vital, mathematical modeling serves as an indispensable tool for elucidating the underlying physics and predicting behavior under untested conditions. The existing modeling landscape can be broadly classified into three paradigms: First, (a) Fickian diffusion models [5] simplistically attribute solvent transport to concentration gradients. In addition to Fickian diffusion, recent studies on selective ion transport in polymeric and functionalized membrane systems have highlighted the importance of molecular-scale pathways, chemical specificity, and ion–channel interactions (e.g., in polyamide nanofiltration membranes and crown-ether grafted channels) [6,7]. Second, (b) non-Fickian models [8,9,10,11,12] incorporate viscoelastic effects of the polymer network. Finally, (c) collective diffusion models [13,14], pioneered by Tanaka et al., reconceptualize gel deformation as a network motion driven by stress gradients. The last of the three successfully establishes a correlation between the characteristic swelling time, the cooperative diffusion coefficient, and the gel dimension, with subsequent extensions by Li and Tanaka [14] accommodating gels of arbitrary shape.

**Figure 1:**
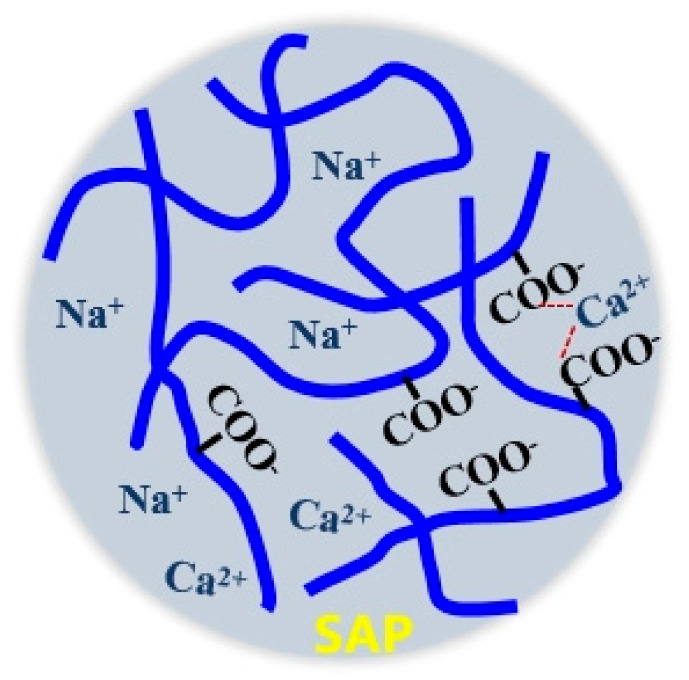
Ion exchange effects between Ca^2+^ and Na^+^.

However, a pivotal physicochemical process, ion exchange, is frequently marginalized in these kinetic models. This is a significant oversight, particularly concerning interactions with the multivalent cations (e.g., Ca^2+^, Al^3+^) ubiquitous in real-world environments (see Figure 1). The ubiquity of Ca^2+^ in tap water, biological fluids, and cementitious environments makes understanding its specific interaction with SAPs a problem of paramount practical importance. In many of these environments, the presence of divalent cations, especially Ca^2+^, dramatically alters SAP performance [15,16]. For example, Ca^2+^ is abundant in cementitious pore solutions used for internal curing, in hard water encountered during hygiene product use, and in physiological fluids relevant to biomedical systems. In polyacrylate SAPs, Ca^2+^ binds to carboxylate groups (–COO^−^), forming ionic crosslinks that stiffen the network and drive desorption. This ion-exchange process is both chemically and mechanically coupled: ion uptake locally increases the elastic modulus, while deformation alters the porosity and diffusion pathways that govern further ion transport.

Although SAPs can be viewed as ion-exchange resins, and classical ion-exchange kinetics models are well-established [17,18,19,20], a critical divergence exists: conventional resins typically undergo negligible volume change, whereas SAPs experience profound deformation during ion exchange [21,22,23,24,25,26]. This deformation dynamically alters the transport pathways and properties within the polymer, thereby influencing subsequent ion exchange. Conversely, the influx of multivalent ions can dramatically alter the elastic modulus of polymer through the formation of ionic crosslinks [27,28]. The existing models often treat ion transport and swelling separately, or neglect the dramatic volume changes of SAPs during ion exchange. Moreover, a key parameter governing the coupling strength is the ion dissociation degree k, i.e., the fraction of Ca^2+^ that remains free versus bound to the polymer network. This dissociation degree determines how many ions participate in diffusion and how many contribute to network stiffening, yet it is rarely explicitly considered in transport–deformation models. The quantitative description of this coupled chemo-mechanical process remains an open challenge, limiting our predictive capability for SAP performance in complex ionic media.

In response to this gap, our work introduces a novel coupled model that explicitly integrates Ca^2+^ transport, described by Fickian diffusion, with SAP deformation dynamics, which are governed by the elastic wave equation. The primary objectives of this study are threefold: (1) to establish a theoretical framework that captures the essential feedback between finite strain deformation and ion transport; (2) to develop a robust numerical scheme to solve the coupled system and uncover the emergent, non-intuitive phenomena arising from this interaction; and (3) to rigorously validate the model against experimental data and elucidate the impact of key parameters, including SAP particle size and solution concentration. This work aims to transition from a phenomenological understanding to a predictive, mechanism-based framework, ultimately guiding the rational design of SAPs for targeted applications in complex ionic environments.

## 2. Results and Discussion

### 2.1. The Effect of SAP Size in Deionized Water

This section mainly presents the numerical simulation results for R75-0Ca, R200-0Ca and R300-0Ca (with different sizes, without Ca^2+^ ion transport). The main outputs of the model are displacement, elastic modulus, porosity and volumetric strain. Since the spatial and temporal distributions of the latter three quantities are similar, displacement and elastic modulus were chosen as representative output parameters. The times selected are 90 s, 300 s and 690 s after SAP water absorption, and the space is taken from the surface of dry, spherical SAP at 10 μm, 30 μm and 70 μm.

#### 2.1.1. Elastic Modulus Evolution

**Figure 2:**
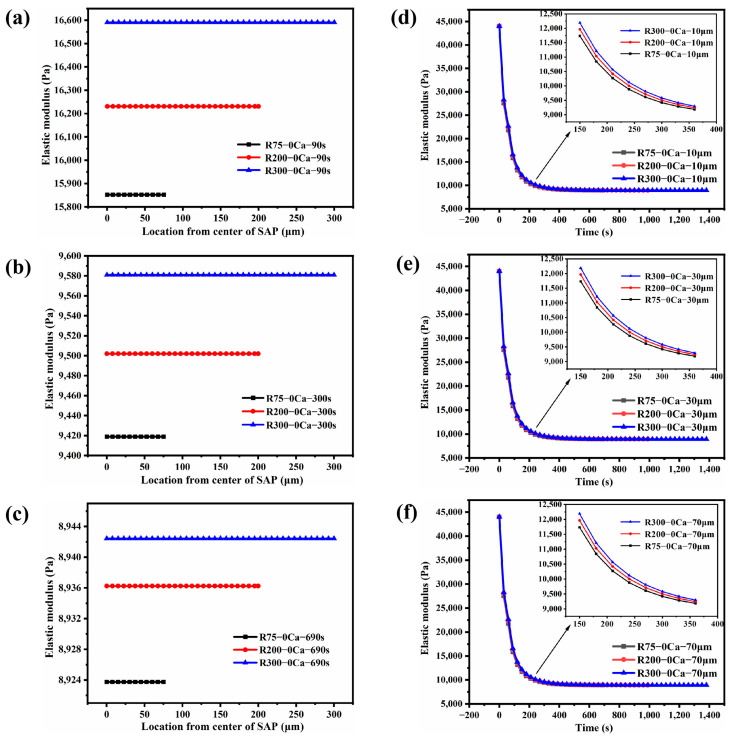
The spatial and temporal distribution of the elastic modulus of spherical SAP with three dry particle sizes (with radii of 75 μm, 200 μm and 300 μm) in deionized water: (**a**–**c**) spatial distribution; (**d**–**f**) temporal distribution.

**Figure 3:**
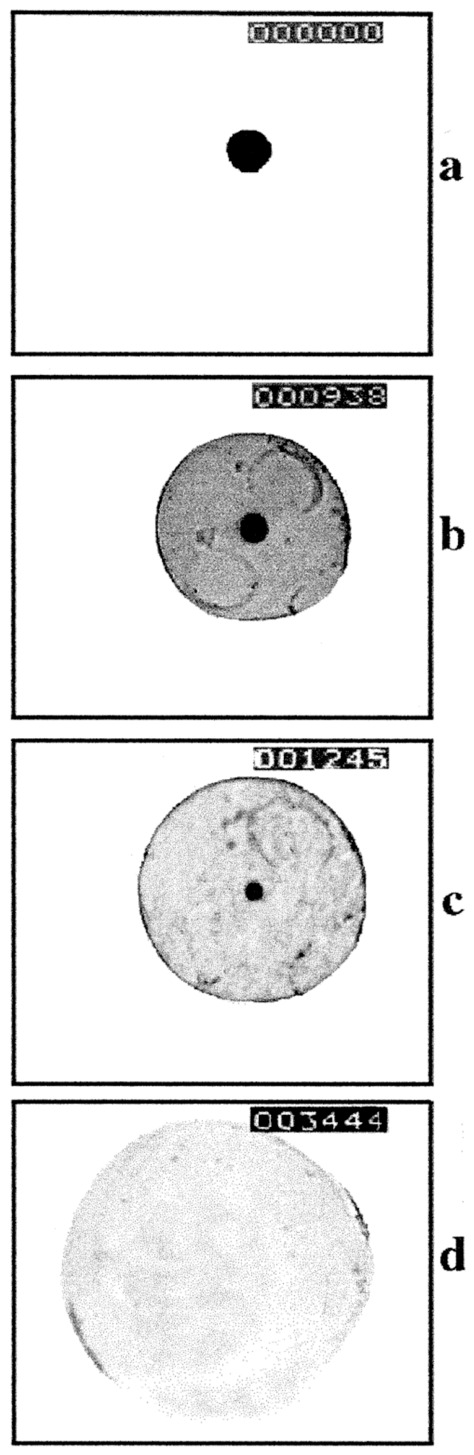
Actual photograph of SAP swelling upon water absorption in deionized water (and exhibiting almost no moisture gradient). Time of swelling is (**a**) 0, (**b**) 0.5, (**c**) 0.7 and (**d**) 2.2 min [29].

**Figure 4:**
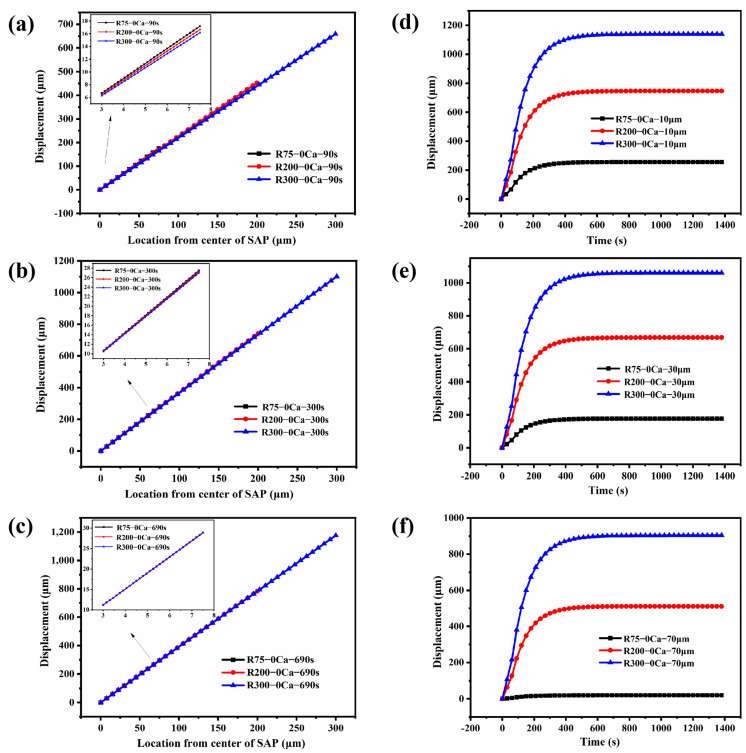
The spatial and temporal distribution of the displacement for spherical SAP with three dry particle sizes (with radii of 75 μm, 200 μm and 300 μm) in deionized water: (**a**–**c**) spatial distribution; (**d**–**f**) temporal distribution.

The spatiotemporal evolution of the elastic modulus (*E*), governed by its porosity (*ϕ*)-dependent constitutive relationship in Equation (26), reveals fundamental aspects of the swelling process in the absence of ion exchange. As predicted, the modulus exhibits an inverse correlation with porosity: increasing water absorption (higher *ϕ*) leads to a plasticization of the polymer network, resulting in a diminished Young’s modulus. The numerical results, depicted in Figure 2, elucidate three key patterns:

(1) Spatial uniformity of the modulus: The spatial uniformity of the elastic modulus is consistent with our model assumption of instantaneous water transport (Figure 2a–c); this consistency does not constitute independent validation but rather shows internal coherence. This simulated result confirms that no significant gradient in water content develops spatially, which is also experimentally observed (see Figure 3). The modulus homogeneity, therefore, is not merely a result but a manifestation of the premise that the polymer network hydrates uniformly and rapidly, leading to a state of near-instantaneous local equilibrium in water distribution.

(2) Temporal softening dynamics: The monotonic decline of the elastic modulus with time at any spatial location underscores the progressive plasticization of the SAP. This temporal softening is not a simple linear process but is intrinsically linked to the kinetics of water ingress. The swelling-induced expansion continuously increases the porosity, which in turn reduces the modulus. The stabilization of the modulus coincides with the attainment of absorption equilibrium, marking a transition from a kinetic to a thermodynamic state in which the network and solvent forces are balanced.

(3) Size-dependent kinetics and the path to equilibrium: The influence of particle size on the modulus evolution (Figure 2d–f) highlights the role of diffusional path length in governing the swelling kinetics. Larger particles, with their longer characteristic diffusion times, absorb water more slowly. Consequently, at any identical time point before equilibrium, a larger SAP particle has a lower average water content (lower *ϕ*) than a smaller one, resulting in a systematically higher modulus across its volume. This suggests a kinetically controlled stiffening effect, which is a model prediction rather than a directly observed phenomenon. The convergence of the equilibrium modulus values across all particle sizes is a significant finding, affirming that the final swollen state is a material property independent of geometry. This aligns well with the experimental observation of identical maximum absorbency (Figure 4), demonstrating that the ultimate degree of hydration and network expansion is governed by the polymer–solvent interaction parameters, not by the initial particle dimensions.

#### 2.1.2. Displacement Field

Figure 4 delineates the spatiotemporal evolution of the radial displacement field for SAPs of varying sizes (R75-0Ca, R200-0Ca, and R300-0Ca) during swelling in deionized water. The simulation results uncover fundamental mechanical responses governed by the symmetry and constitutive behavior of the system.

(1) Linearity of the displacement field: The spatially linear profile of radial displacement at any given time (Figure 4a–c) is a direct kinematic consequence of uniform, isotropic swelling under spherical symmetry. This linearity is not merely a numerical result but a fundamental solution to the deformation kinematics for a homogeneously expanding sphere. The underlying driver for this pattern is the spatially uniform elastic modulus (Figure 2), which ensures that the resistance of material to deformation is identical at all points. Consequently, the applied driving force for swelling (the osmotic pressure) generates a strain field that is homogeneous, directly manifesting as a linear displacement from the center (where symmetry dictates u = 0) to the unconstrained surface.

(2) Temporal evolution and swelling kinetics: The monotonic increase of displacement at all locations until stabilization (Figure 4d–f) provides a mechanical representation of the absorption kinetics, as quantified in Figure 4. Each point in the displacement–time curve corresponds to the progressive ingress of water and the consequent volumetric expansion. The stabilization of displacement marks the attainment of thermodynamic equilibrium between the elastic retraction force of the polymer network and the osmotic swelling pressure.

(3) Particle size effect: While the absolute displacement is consistently larger for bigger SAPs at any comparable time and spatial coordinate (e.g., comparing u at r = 10 μm for different sizes), this reflects a difference in absolute dimensions rather than material behavior. A more insightful metric is the volumetric strain, which is comparable across sizes at equilibrium. The convergence of the displacement profiles towards similar relative expansions as equilibrium is approached underscores a critical finding: the final swollen state is governed by material thermodynamics, not initial size. The larger initial displacements in bigger particles are a kinetically controlled artifact, as they have a longer path to the same relative equilibrium state defined by the polymer–solvent interaction parameter.

### 2.2. Effect of SAP Size in Solution Containing 20 mM Ca^2+^

This section mainly shows the numerical simulation results of groups R75-20Ca, R200-20Ca and R300-20Ca. Additionally, the coupled results of SAP deformation and Ca^2+^ ion transport are the focus of the entire model, which is discussed carefully here. The simulation outputs are free ion concentration, the concentration of crosslinked Ca^2+^ ions, diffusion coefficient, displacement, elastic modulus, porosity, and volumetric strain. Likewise, since the spatio-temporal distributions of some quantities are similar, only free ion concentration, volumetric strain, and elastic modulus are shown in this section. As for time, 1.5 min, 75 min and 301 min (for volumetric strain, there is no 301 min, but 10 min is shown) after water absorption were selected, and as for space, the 10 μm, 30 μm and 70 μm distances from the SAP surface were shown.

#### 2.2.1. Free Ca^2+^ Ion Concentration

**Figure 5:**
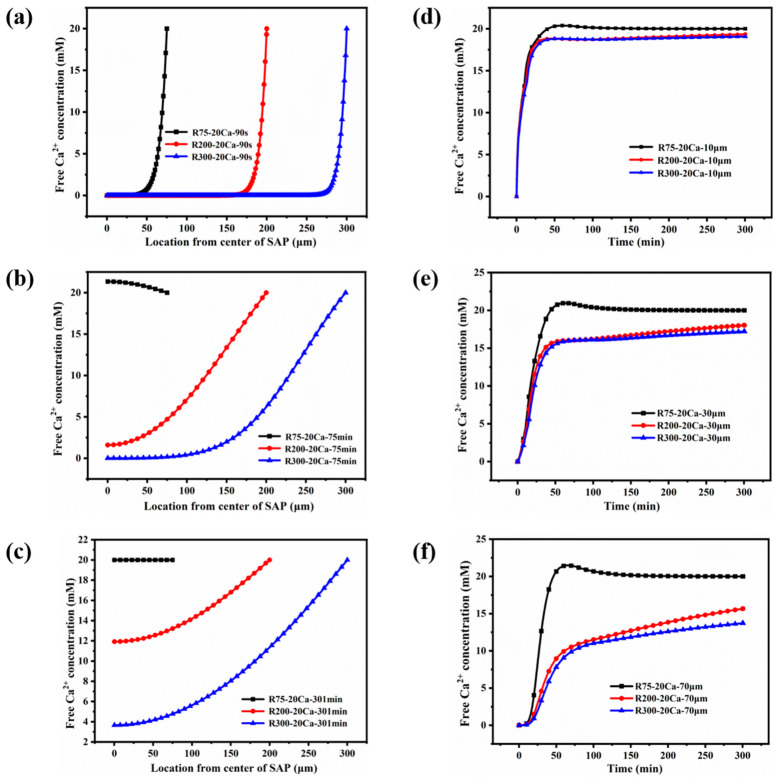
The spatial and temporal distribution of free Ca^2+^ ion concentration of spherical SAP with three dry particle sizes (with radii of 75 μm, 200 μm and 300 μm) in solutions containing 20 mM Ca^2+^: (**a**–**c**) spatial distribution; (**d**–**f**) temporal distribution.

Figure 5 shows the spatial and temporal distribution of free Ca^2+^ ion concentration for groups R75-20Ca, R200-20Ca and R300-20Ca. The results of numerical simulation mainly show the following rules.

For the smallest-size SAP (with a radius of 75 μm), the model outputs a novel result. At any spatial position of the SAP, the temporal distribution of the free Ca^2+^ ion concentration has a peak value (see R75-20Ca of Figure 5d–f), and the peak exceeds the boundary concentration (20 mM). In addition, from the point of view of time, it is divided into early and late stages. In the early stage of water absorption (Figure 5a), the spatial distribution of the free Ca^2+^ ion concentration is a normal gradient distribution (from low to high), while in late stage (Figure 5b), the spatial distribution of the concentration is reverse (from high to low). The subsequent concentration (without any effect of deformation on ion transport) gradually becomes equal everywhere (no gradient, Figure 5c) and equal to the boundary concentration.

**Figure 6:**
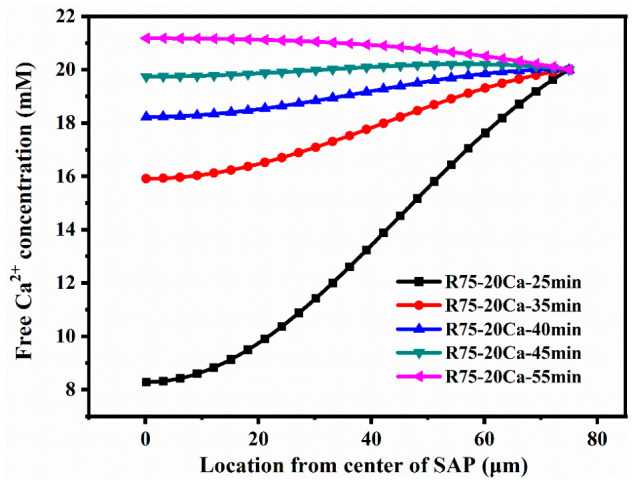
The spatial distribution of free Ca^2+^ ion concentration at more detailed times (SAP size: a radius of 75 μm; Selected times: 25 min, 35 min, 40 min, 45 min and 55 min).

**Figure 7:**
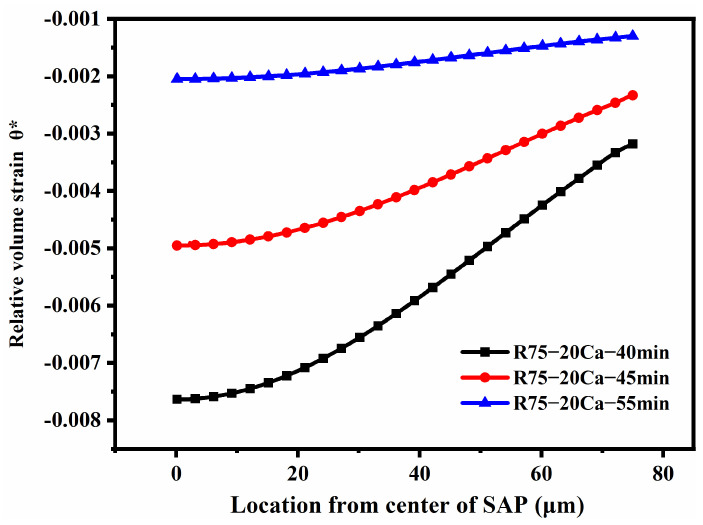
The spatial distribution of relative volume strain ($\theta^{*}$) at 40 min, 45 min and 55 min (SAP size: a radius of 75 μm).

To clearly reflect the reverse process of concentration gradient, the spatial distribution of free Ca^2+^ ion at more detailed time is shown in Figure 6. The novel simulation results are just the coupled effects of ion transport and deformation. To explain the results further, Figure 7 illustrates the spatial distribution of relative volume strain ($\theta^{*}$) at 40 min, 45 min and 55 min. It is clear that, compared to that at the surface of SAP, the absolute value of $\theta^{*}$ is bigger near the center of SAP; namely, there is a bigger decrease of volume. Consequently, according to Equation (30), a higher ion concentration ($C_{B}$, even more than boundary concentration 20 mM) is generated at the center of SAP when the ion concentration near center is close to 20 mM.

As the particle size of the SAP increases, the effect of (1) will weaken and disappear. The main reason for this may be attributed to two points. One is that during desorption deformation of SAPs, the larger-sized SAPs have a lower Ca^2+^ ion concentration near the center of the SAP, owing to the longer transport distance. The other is that larger-sized SAPs have a lower desorption rate, causing a smaller $\theta^{*}$.

To verify that the predicted free Ca^2+^ concentration overshoot (exceeding the boundary value) is not a numerical artifact, we performed three consistency checks (see Figures S3 and S4 in the Supplementary Materials): (1) total Ca^2+^ mass in the SAP (free + crosslinked) was computed using Equation (35) and confirmed to be conserved within 10^−5^% over the entire simulation; (2) the overshoot persisted under mesh refinement (h = 0.375, 0.1875, and 0.125 μm) and (3) time-step reduction (τ = 0.0005, 0.001, and 0.002 s). These tests support the physical origin of the overshoot, which arises from the volume contraction near the particle center concentrating free ions, as described in Equation (21).

#### 2.2.2. Elastic Modulus

Figure S9 in the Supplementary Materials shows the space–time distribution of elastic modulus for R75-20Ca, R200-20Ca and R300-20Ca. The results of the numerical simulation mainly show the following rules. As concluded in Section 2.2.1, the concentration of free Ca^2+^ ions presents a spatial gradient distribution during deformation. According to Equation (14), therefore, the cross-linked Ca^2+^ ions must have a distribution similar to that of free Ca^2+^ ions. Further, based on Equation (26), the elastic modulus of SAP depends on the SAP porosity and the concentration of cross-linked Ca^2+^ ions. As distinct from that in deionized water, the elastic modulus of SAP in solution containing 20 mM Ca^2+^ is not only affected by porosity, but also by the concentration of cross-linked Ca^2+^ ions. In other words, the transport of Ca^2+^ ions must be taken into account during deformation, which makes the spatial distribution of elastic modulus present a gradient (see Figure S1a–c in the Supplementary Materials). Similarly, the special time distribution (a peak) of the concentration of free Ca^2+^ ions for the smallest-size SAP (with a radius of 75 μm) leads to a corresponding time distribution of the elastic modulus (see Figure S1d–f in the Supplementary Materials).

#### 2.2.3. Volume Strain

**Figure 8:**
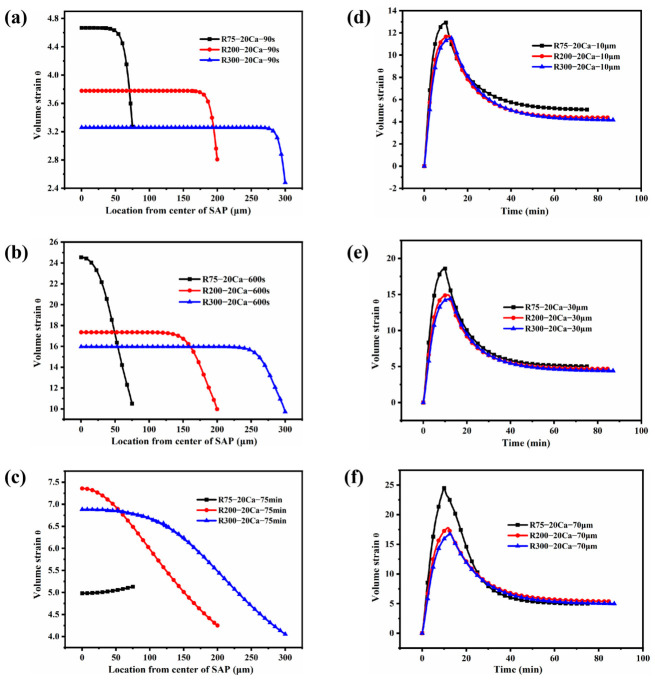
The spatial and temporal distribution of volume strain of spherical SAP with three dry particle sizes (with radii of 75 μm, 200 μm and 300 μm) in solutions containing 20 mM Ca^2+^: (**a**–**c**) spatial distribution; (**d**–**f**) temporal distribution.

Figure 8 delineates the spatiotemporal distribution of volumetric strain for SAPs in Ca^2+^-containing solutions (R75-20Ca, R200-20Ca, and R300-20Ca), revealing a mechanical state fundamentally shaped by the coupling process. The results demonstrate a constitutive relationship between strain and modulus, and a spatial heterogeneity absent in pure water.

**Figure 9:**
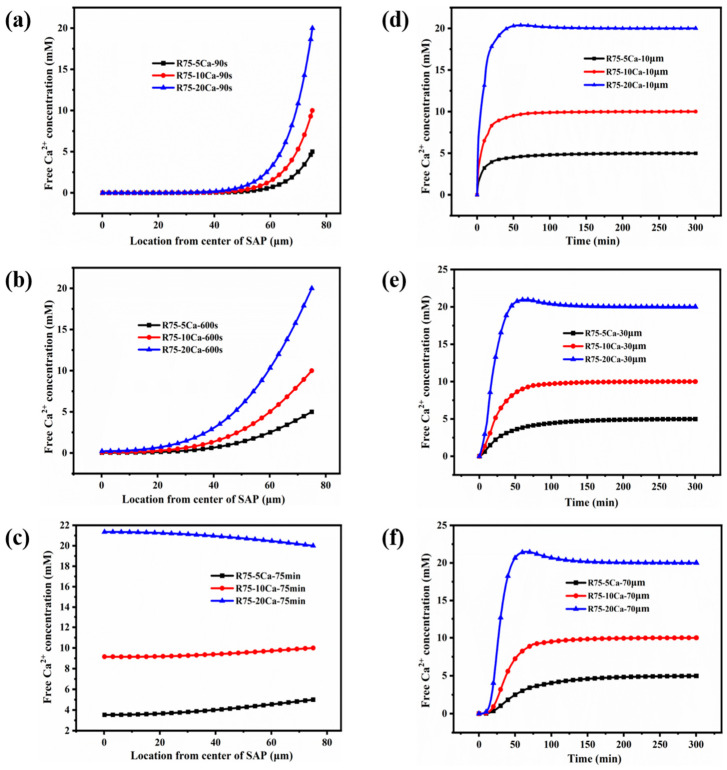
The spatial and temporal distribution of free Ca^2+^ ion concentration of spherical SAP (with a radius of 75 μm) in solutions containing three kinds of Ca^2+^ concentration (5 mM, 10 mM and 20 mM): (**a**–**c**) spatial distribution; (**d**–**f**) temporal distribution.

(1) The constitutive antagonism between strain and modulus: The observed inverse correlation between volumetric strain (Figure 8) and elastic modulus (Figure S1) is a direct manifestation of the stress equilibrium within the swelling polymer network. The spatial gradient of Ca^2+^ ions dictates a corresponding gradient in ionic crosslink density ($C_{RB}$). Regions with higher crosslinking (and thus a higher modulus, see Equation (26)) exhibit greater resistance to expansion, resulting in suppressed local swelling (lower volumetric strain, see Figure 8a–c). Conversely, regions with lower crosslink density remain more compliant and can undergo greater expansion. This strain–modulus antagonism creates an internally structured material in which a “soft” domain swells significantly, adjacent to a “stiff,” less-swollen domain, and both are orchestrated by the diffusing ion.

(2) Ion-mediated programming of internal strain: The emergence of a volumetric strain gradient is a hallmark of the coupling effect. Unlike the homogeneous strain field in deionized water (a result of uniform modulus), the presence of Ca^2+^ enables the “programming” of an internal strain field via diffusion. The ion concentration gradient serves as a template that, through its effect on the local elastic modulus, is transcribed into a corresponding mechanical strain gradient. This phenomenon underscores that the internal state of the SAP is not merely a function of external boundary conditions but is dynamically shaped by the evolving internal chemical field.

(3) Particle size effect on strain localization: The attenuation of the volumetric strain peak with increasing particle size (Figure 9d–f) can be attributed to two scaling effects. First, the diffusional time scale increases with the square of the particle radius, leading to a slower and more diluted accumulation of Ca^2+^ in the core of larger particles. This results in a weaker and more spatially homogeneous crosslinking gradient. Second, the weaker desorption response in larger particles generates a smaller overall driving force for volume change. The combined effect is a less pronounced coupling feedback loop, leading to diminished strain localization and a more uniform, albeit smaller, volumetric strain distribution throughout the particle.

### 2.3. Effect of Ca^2+^ Concentration

This section mainly shows the numerical simulation results for groups R75-5Ca, R75-10Ca and R75-20Ca. Similarly, only the spatio-temporal distribution of free ion concentration, volume strain and elastic modulus are shown in this section. The selection of time and space elements is consistent with that in Section 2.2.

#### 2.3.1. Free Ca^2+^ Ion Concentration

Figure 9 delineates the spatial and temporal distribution of free Ca^2+^ ion concentration under varying external concentrations (R75-5Ca, R75-10Ca, and R75-20Ca), highlighting a critical concentration threshold for the emergence of strong coupling dynamics.

(1) Concentration-driven ion uptake: The monotonic increase in internal free Ca^2+^ concentration with rising external concentration, observed across all spatial locations and time points, is governed by the fundamental chemical potential gradient. This establishes a direct link between the boundary condition and the internal state, confirming the model’s ability to simulate Fickian transport under a varying driving force.

(2) The threshold for emergent coupling phenomena: The absence of the novel concentration overshoot and gradient inversion (detailed in Section 2.2.1) in the R75-5Ca and R75-10Ca groups is a pivotal finding. It indicates that a critical concentration threshold must be exceeded to activate the strong feedback loop responsible for these non-intuitive results. Below this threshold, the system behavior is dominated by standard diffusion and swelling, absent significant chemo-mechanical feedback.

(3) Mechanism of threshold activation: The underlying mechanism for this threshold behavior lies in having sufficient ionic crosslinking to induce a pronounced desorption response. At lower concentrations (5–10 mM), the internal concentration of crosslinked Ca^2+^ ($C_{RB}$) remains too low to significantly increase the elastic modulus and trigger substantial desorption. Consequently, the volumetric strain remains positive and relatively uniform, failing to generate the strong, localized contraction (negative $\theta^{*}$) required to concentrate the ions and produce the overshoot phenomenon. The weaker desorption rate observed experimentally is thus not merely a correlate but the manifestation of this sub-critical crosslinking state. The full coupling cycle—in which transport alters mechanics, which in turn retroacts on transport—only becomes self-sustaining and observable when the external ion supply is sufficient to drive the internal crosslink density past a critical point.

#### 2.3.2. Elastic Modulus

The spatio-temporal distribution of the elastic modulus for SAPs under varying Ca^2+^ concentrations (R75-5Ca, R75-10Ca, and R75-20Ca), as shown in Figure S2 in the Supplementary Materials, provides a quantitative map of how ionic crosslinking reinforces the polymer network. The analysis reveals a dose-dependent stiffening effect with profound implications for the network structure.

(1) Dose-dependent network reinforcement: The systematic increase in elastic modulus with external Ca^2+^ concentration, observable throughout the SAP volume and across time, is a direct signature of progressive ionic crosslinking. Each divalent Ca^2+^ ion can bridge two anionic carboxylate groups (–COO^−^) on the polymer backbone, introducing additional topological constraints that reduce chain mobility and enhance the network’s resistance to deformation. The gradient in modulus observed at a given time further reflects the diffusion-limited front of crosslink formation, painting a dynamic picture of a self-stiffening material.

(2) The mechanistic basis of limited modulus enhancement: The relatively modest enhancement of the elastic modulus by Ca^2+^, especially when contrasted with the potent effects of Cu^2+^ or Al^3+^, as noted in the literature, is not a model shortcoming but a reflection of the underlying ionic bond strength and coordination chemistry. The crosslinks formed by Ca^2+^ are predominantly electrostatic in nature, which are weaker and more dynamic (prone to breaking and re-forming) compared to the stronger, more covalent-like coordination bonds that transition metal ions like Cu^2+^ can form with carboxyl groups. This results in Ca^2+^ acting as a moderate crosslinker, increasing the modulus but failing to induce the orders-of-magnitude stiffening characteristic of stronger coordinative crosslinkers. This distinction is crucial for accurately predicting SAP behavior in different ionic environments.

(3) Constitutive Implications for the model: The observed trend validates the linear assumption between $C_{RB}$ and modulus in Equation (26) for the Ca^2+^ system. The model successfully captures that the mechanical state is a superposition of the baseline porosity-dependent modulus and a linearly scaled contribution from ionic crosslinks. The fact that the modulus enhancement is very limited is quantitatively encoded in the fitted value of the coefficient *w*, which would be substantially larger for ions like Cu^2+^ or Al^3+^. This underscores the model’s potential to generalize across different ion types by calibrating this key parameter.

#### 2.3.3. Volume Strain

This section shows the spatio-temporal distribution of the volume strain for groups R75-5Ca, R75-10Ca and R75-20Ca.

**Figure 10:**
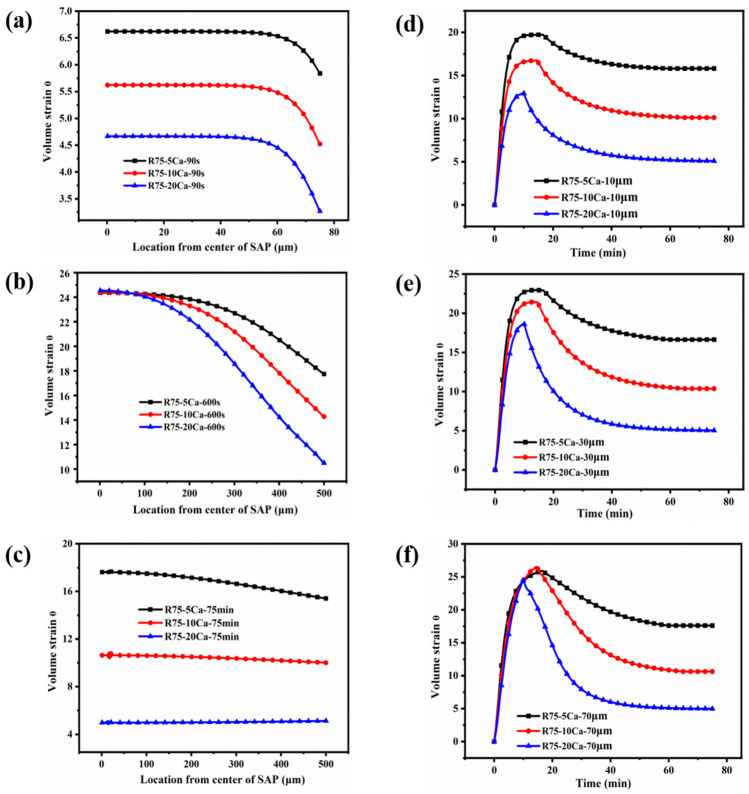
The spatial and temporal distribution of volume strain of spherical SAP (with a radius of 75 μm) in solutions containing three kinds of Ca^2+^ concentration (5 mM, 10 mM and 20 mM): (**a**–**c**) spatial distribution; (**d**–**f**) temporal distribution.

With the increase of Ca^2+^ ion concentration in solution, the volume strain of SAP becomes smaller in any space and at any time. Likewise, the spatio-temporal distribution of volume strain is opposite to that of elastic modulus (see Figure 10).

### 2.4. Effect of Dissociation Degree

The dissociation degree of Ca^2+^ (k, see Equation (14)) is an important parameter in our model, and significantly influences the coupling dynamics. k is dependent on the type of resin and the free ions. 

(1) Effect of resin type on k: For divalent Ca^2+^ ions, the k in strong acid type resins (for example, the resin containing –(SO_3_)_2_Ca) is generally 0.008 [30], while for weak acid resins (for example, the resin containing –(COO)_2_Ca), the range of k is 0.002 to 0.008 [31].

(2) Effect of free ion type on k: For the polyacrylic acid type resin, the dissociation degree of the monovalent Na^+^ ions (k = 0.04) is much higher than that of the divalent Ca^2+^ (k = 0.002–0.008).

**Figure 11:**
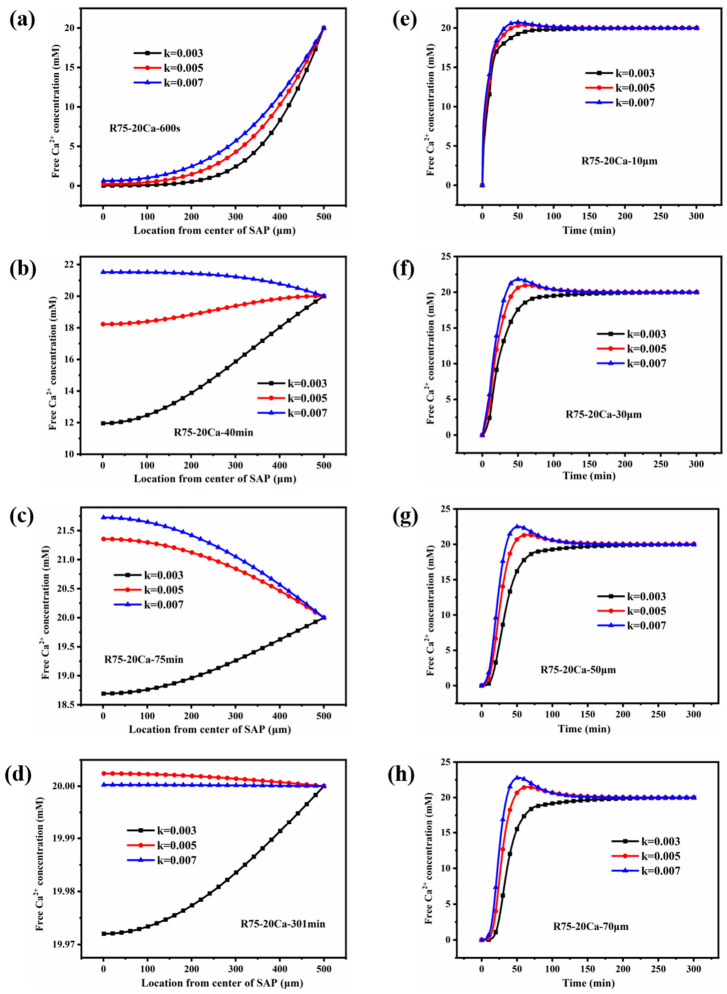
The spatial and temporal distribution of free Ca^2+^ ions for R75-20C, a group with different k (0.003, 0.005 and 0.007): (**a**–**d**) spatial distribution; (**e**–**h**) temporal distribution.

In Section 2.1, Section 2.2 and Section 2.3, k was consistently 0.005. To study the effect of k on the simulation results, the spatial and temporal distribution of free Ca^2+^ ions for the R75-20Ca group with different k (0.003, 0.005 and 0.007) is shown in Figure 11. The times selected are 10 min, 40 min, 75 min and 301 min after SAP water absorption and the space comprises distances of 10 μm, 30 μm, 50 μm and 70 μm from the surface of the dry, spherical SAP. Two critical conclusions are summarized as follows. The novel simulation result discussed in Section 2.2.1 begins to appear earlier as k becomes larger (see Figure 11b,c). A possible reason is that a higher k means a higher free Ca^2+^ ion concentration in SAP, which makes the free Ca^2+^ ion concentration near the center of the SAP reach 20 mM earlier. A higher k generates a higher peak of the concentration of free Ca^2+^ ions in the temporal distribution. One possible explanation is as follows. Based on the conclusion of (1), a higher k makes the free Ca^2+^ ion concentration reach 20 mM earlier, corresponding to an earlier desorption time. Further, according to Figure 7, an earlier desorption has a higher relative volume strain ($\theta^{*}$), which changes the ion concentration more dramatically based on Equation (30). As a result, a higher peak appears for a higher k.

The dissociation degree k is influenced by several environmental factors not explicitly modelled here [32]. The pH affects the protonation of carboxylate groups (pKa ~4–5); at a low pH, protonation reduces available binding sites, thereby decreasing effective crosslinking. Ionic strength screens electrostatic interactions, altering both Donnan equilibrium and Ca^2+^ binding affinity. Temperature affects the binding equilibrium constant and diffusion coefficients. In the current phenomenological framework, these effects can be incorporated by parameterizing k, $D_{r}$, and w as functions of these variables—an extension left for future work.

### 2.5. Verification

To quantitatively assess the model’s predictive capability, we compared the simulated total Ca^2+^ uptake with experimental measurements. The total amount of Ca^2+^ ions (including both free and crosslinked Ca^2+^ ions) trapped by a single spherical SAP can be calculated using Equation (1).

(1) $$A\left(t\right)=\sum_{i=1}^{M}C_{all}\left(r_{i},t\right)4\pi i^{2}h^{3}(t)\;$$

where A(t) is the total amount of Ca^2+^ ions trapped by SAP at moment t, $C_{all}(r_{i},t)$ is the total concentration of Ca^2+^ ions at position $r_{i}$ at moment t, i is the i-th node in space, M is the total number of nodes in space and h(t) is the spatial step at moment t.

**Figure 12:**
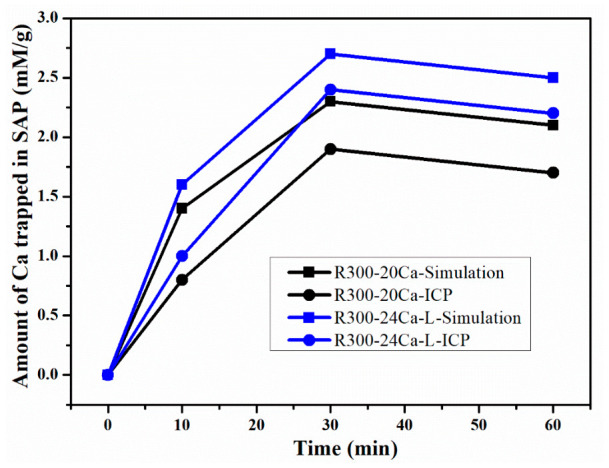
Comparison of simulation and experimental data for amount of Ca^2+^ uptake in SAP at different times, for R-300-24Ca-L in reference [29], and for R-300-20Ca in this paper.

The total amounts of Ca^2+^ ions for R300-20Ca in this paper, as obtained by ICP, and for R300-24Ca-L, as obtained in reference [29], are compared with those obtained by simulations and Equation (1); the result is shown in Figure 12.

Figure 12 compares simulated and experimentally measured total Ca^2+^ uptake for R300-20Ca and R300-24Ca-L. All model parameters used for this prediction were determined independently: k = 0.005 (from the range in the literature for polyacrylate-Ca^2+^ [33,34]), $D_{r}$ (calculated from ion self-diffusivities in water with porosity correction), E_0_ and $\emptyset_{0}$ (from dry SAP characterization), $\tau_{a}$ and $\tau_{r}$ (fitted from absorption/desorption curves), and w (regression from modulus data in Ref. [27]). No parameters were adjusted to match the Ca^2+^ uptake data. The simulation captures both the initial rise and the non-monotonic trend (a subsequent decrease is due to ion expulsion during desorption) with a mean relative error of 8%, providing quantitative support for the model’s coupled transport–deformation mechanism.

We acknowledge that the validation presented here is based on bulk Ca^2+^ uptake and macroscopic radius evolution. Direct measurement of internal Ca^2+^ concentration profiles (e.g., via confocal Ca^2+^-indicators or Raman mapping) and local deformation (e.g., micro-CT) is not yet available. Such experiments are essential to fully verify the model’s predictive capability and are planned for future studies.

## 3. Conclusions

This study developed and numerically implemented a phenomenological coupled model integrating Fickian Ca^2+^ diffusion with elastic deformation dynamics for spherical polyacrylate SAP particles. The main coupling mechanisms of deformation-dependent diffusivity and Ca^2+^-crosslink-enhanced modulus were expressed via a variable-coefficient partial differential system solved by finite differences. The model was validated against total Ca^2+^ uptake measured by ICP for two independent datasets, with a mean relative error of 8%. The model predicts the following chemo-mechanical coupling phenomena:

(1) For small SAP particles (75 μm) in 20 mM Ca^2+^, the free Ca^2+^ concentration transiently overshoots the external boundary value by 15% and later exhibits a spatial concentration gradient inversion.
(2) A threshold external Ca^2+^ concentration (15–20 mM) is required to activate this strong feedback loop.
(3) Ionic crosslinking induces spatially non-uniform elastic modulus and volumetric strain fields, with the mechanical state being “programmed” by the diffusing ion profile.

These internal-field phenomena are model predictions that await direct experimental validation via spatially resolved techniques.

The parameter k = 0.005 was found to be representative for Ca^2+^ in polyacrylate SAPs, and the model results are sensitive to its value, indicating the importance of accurate dissociation data. Future work will extend the model to include explicit water transport, finite-strain mechanics, and local-binding isotherms, and will pursue direct imaging validation of the predicted internal fields.

## 4. Materials and Methods

### 4.1. Materials

The SAP used was a commercial polyacrylate SAP (Shenyang Corestone, Shenyang, China) consisting of crosslinked sodium polyacrylate hydrogel with carboxylate functional groups. Spherical particles with nominal radii of 75 ± 20 μm, 200 ± 20 μm, and 300 ± 20 μm were sieved. The particle size distribution of the 75 μm SAP is presented in Figure S3. The reagents were NaCl (AR) and Ca(OH)_2_ (AR). All experiments were conducted at a controlled temperature of 25 ± 1 °C.

Comparing the experimental conditions of the R300-24Ca-L dataset in Ref. [26] with our own measurements, as to SAP composition, both studies used polyacrylate-type SAPs with carboxylate functional groups, ensuring comparable ion-binding chemistry. For solution, the literature study used 24 mM Ca^2+^ (as CaCl_2_) with background Na^+^, while we used 20 mM Ca^2+^ (as Ca(OH)_2_) with 360 mM Na^+^. We have now noted that the small difference in Ca^2+^ concentration and counter-anion may cause minor quantitative deviations, but the underlying Ca^2+^-carboxylate crosslinking mechanism remains identical.

### 4.2. Absorption and Desorption Measurements

The main variables considered in this model were Ca^2+^ ion concentration and SAP particle size. The specific concentrations of Ca^2+^ and Na^+^ cations (marked as $C_{Ca}$ and $C_{Na}$) are shown in Table 1. The experiment group named R75-20Ca represents the SAP with a radius of 75 μm in solution containing 20 mM Ca^2+^. The remaining groups in Table 1 were marked in a similar manner. The last group, R300-24Ca-L, is from reference [26].

The ab- and desorption curves for all experiment groups (see Table 1) were determined using the classical tea bag method [35]. The tea bag method was carried out, in detail, as follows. About 0.3 g of spherical SAP (exact mass $m_{1}$) is placed into a tea bag (mass $m_{2}$) that has been pre-wetted in solution. The tea bag is immersed in a beaker containing 500 mL of the test liquid. The beaker is tightly wrapped in plastic wrap to avoid carbonization. During the weighing process, the plastic foil is removed only for a short period of time. After the pre-determined period of time, the tea bag containing the SAP is lifted and weighed (mass $m_{3}$). Before weighing, place the tea bag on a dry cloth and quickly remove any excess water from the surface with another dry cloth. In fact, the absorption capacity of SAP is overestimated due to the residual liquid between the swollen SAP particles, when using our method [36]. The absorption capacity of SAP in liquid at different times ($A_{t}$) was calculated using Equation (2). Each curve in the liquid groups is the average of three replicates.

(2) $$A_{t}=\frac{m_{3}-m_{2}{-m}_{1}}{m_{1}}$$

### 4.3. Conversion from Ensemble Uptake to Single-Particle Radius

The method measures ensemble behavior of a particle population rather than the response of a single spherical particle. The nominal particle radii represent average values; polydispersity and particle aggregation may affect the fitted time constants. To convert ensemble mass uptake to single-particle radius evolution, we assumed a monodisperse population and used the average particle volume calculated from the nominal radius. The associated uncertainty is shown in Figures S4 and S5 in the Supplementary Materials.

For the smallest nominal radius of 75 μm, the distribution ranged from 55 μm to 95 μm. Because diffusion time scales with the square of the radius, this spread can affect the inferred kinetics [37]. We therefore performed a sensitivity analysis (see Figure S6 in the Supplementary Materials) in which the model was run with the lower and upper bounds of the size distribution. The predicted concentration overshoot and gradient inversion remained qualitatively present for a smaller size of SAP, although the timing and magnitude varied within 20%. This indicates that the main phenomena are robust to the observed polydispersity.

### 4.4. Total Ca^2+^ Uptake Measurement

For selected groups (R300-20Ca, this study; R300-24Ca-L, Ref. [26]), the total Ca^2+^ content trapped in the SAP was determined by inductively coupled plasma optical emission spectrometry (ICP-OES) after acid digestion.

### 4.5. Boundary Condition of Deformation Equation

The relationship between SAP radius and time is an important boundary condition for the numerical solution of the deformation equation, for which a specific expression (see Equations (4) and (5)) is given through the theoretical model [13,14]. The parameter τ can be determined through fitting the ab- and desorption curves.

#### 4.5.1. Results of Ab- and Desorption Curves

**Figure 13:**
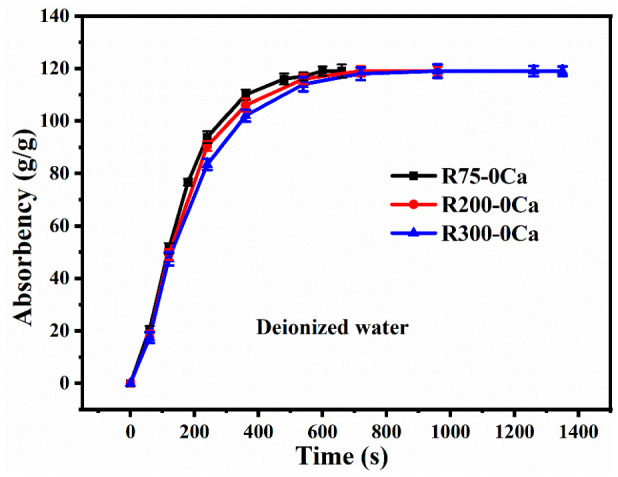
The ab- and desorption curves of R75-0Ca, R200-0Ca and R300-0Ca (SAP size: radii of 75 ± 20 μm, 200 ± 20 μm and 300 ± 20 μm; Solution: Deionized water).

Figure 13 shows the ab- and desorption curves of R75-0Ca, R200-0Ca and R300-0Ca. It can be inferred from Figure 13 that in the deionized water, the larger the SAP particle size, the longer equilibrium time, and the lower absorption speed. Additionally, as can be seen in Figure 4, the maximum absorbency of SAP for three different particle sizes in the deionized water is nearly the same, indicating that SAP size has a negligible effect on the maximum absorbency.

**Figure 14:**
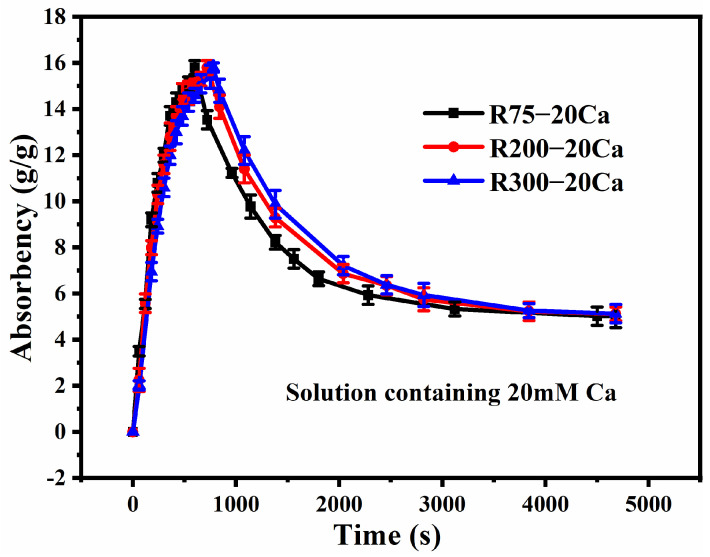
The ab- and desorption curves of R75-20Ca, R200-20Ca and R300-20Ca (SAP size: radii of 75 ± 20 μm, 200 ± 20 μm and 300 ± 20 μm; Solutions: 20 mM Ca).

Likewise, Figure 14 shows the ab- and desorption curves of R75-20Ca, R200-20Ca and R300-20Ca. And the demonstrated law (the larger the particle size, the longer the peak time, and the lower the absorption speed) in Figure 14 is similar to that in Figure 13 before the peak time. The curves in solution containing 20 mM Ca^2+^ ions have an intense desorption, but not in deionized water; this is induced by the ionic crosslinking interaction between Ca^2+^ and –COO^−^ [31,38]. In addition to the direct Ca^2+^–carboxylate crosslinking, other mechanisms contribute to the observed deswelling: the high Na^+^ background (360 mM) compresses the electrical double layer and screens fixed charges on the network; the Donnan equilibrium establishes an osmotic pressure difference; and the release of Na^+^ during ion exchange alters the internal osmotic balance. In the present model, these effects are implicitly captured by the phenomenological parameters: the osmotic driving force is embedded in the fitted boundary radius evolution, and the effective crosslink contribution to the modulus reflects the net mechanical consequence of all ionic interactions.

**Figure 15:**
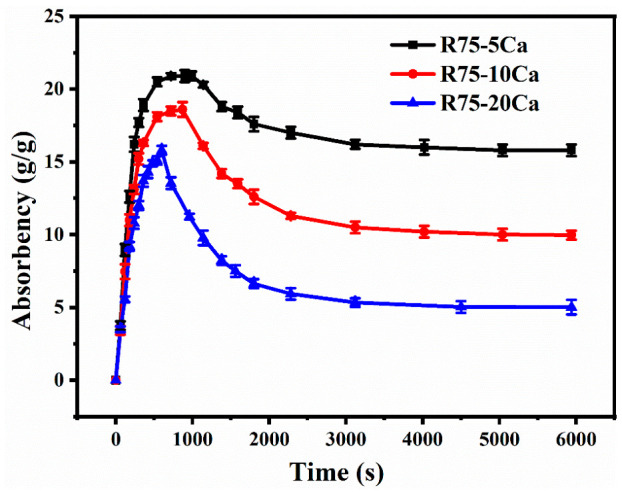
The ab- and desorption curves of groups R75-5Ca, R75-10Ca and R75-20Ca (SAP size: radii of 75 ± 20 μm; Solutions: 5 mM, 10 mM and 20 mM Ca^2+^, respectively).

Figure 15 shows the ab- and desorption curves of groups R75-5Ca, R75-10Ca and R75-20Ca. As can be clearly seen, both the maximum absorbency and the retention capacity of SAP are reduced as the concentration of Ca^2+^ ions in solution increases. The reason for this is that higher Ca^2+^ ion concentration in solution leads to a higher ion-crosslinked extent between Ca^2+^ and SAP, further lowering the maximum absorbency and the retention capacity of SAP.

#### 4.5.2. Fitting Ab- and Desorption Curves

The Tanaka–Fillmore model proposed a characteristic time constant (τ) to describe the ab- and desorption process of SAP, which is expressed in Equation (3):

(3) $$\tau =\frac{r_{l}^{2}}{D_{coop}}$$

where $r_{l}$ is the final radius of the SAP. For the water absorption process, $r_{l}$ is the maximum radius of the SAP, but for the water desorption process, $r_{l}$ is the minimum radius of the SAP. $D_{coop}$ is the co-diffusion coefficient, which generally ranges from 10^−7^ to 10^−6^. According to their theory, the water absorption and desorption curves for SAP are expressed by Equations (4) and (5) respectively.

(4) $$r\left(t\right)=r_{0}+\left(r_{max}-r_{0}\right)\cdot \left(1-e^{-\frac{t}{\tau_{a}}}\right)\;\;\;\;\;\;\;\;\;\;\;\;\;\;\;\;\;\;\;t\leq t_{max}\;$$

(5) $$r\left(t\right)=r_{l}+\left(r_{max}-r_{l}\right)\cdot e^{-\frac{t-t_{max}}{\tau_{r}}}\;\;\;\;\;\;\;\;\;\;\;\;\;\;\;\;\;\;\;\;\;\;\;t>t_{max}$$

where $r_{0}$ is the initial radius of the SAP, $r_{max}$ is the maximum radius of the SAP and $t_{max}$ is the moment corresponding to the maximum radius of the SAP (maximum absorbency). It is not difficult to obtain $r_{0}$, $r_{max}$, $r_{l}$ and $t_{max}$ by measuring the curves. Although τ in Equation (4) ($\tau_{a}$) and Equation (5) ($\tau_{r}$) theoretically can be derived from Equation (3), the co-diffusion coefficient ($D_{coop}$) is influenced by lots of factors [13,14], making it more difficult to quantify $D_{coop}$. Further, the uncertain $D_{coop}$ also makes it difficult to quantify τ. Luckily, we can fit τ through the available ab- and desorption curves using Equations (4) and (5). To fit accurately and conveniently, Equations (4) and (5) are transformed into Equations (6) and (7).

(6) $$\ln\left(1-\frac{r\left(t\right)-r_{0}}{r_{max}-r_{0}}\right)=-\frac{t}{\tau_{a}}\;\;\;\;\;\;\;\;\;\;\;\;\;\;\;\;\;\;\;\;\;\;\;\;\;\;\;\;\;\;\;\;\;\;\;\;\;\;\;\;t\leq t_{max}$$

(7) $$\ln\left(\frac{r\left(t\right)-r_{l}}{r_{max}-r_{l}}\right)=-\frac{t-t_{max}}{\tau_{r}}\;\;\;\;\;\;\;\;\;\;\;\;\;\;\;\;\;\;\;\;\;\;\;\;\;\;\;\;\;\;\;t\leq t_{max}$$

Let $Y_{1}=\ln\left(1-\frac{r\left(t\right)-r_{0}}{r_{max}-r_{0}}\right)$, $X_{1}=t$; $Y_{2}=\ln\left(\frac{r\left(t\right)-r_{l}}{r_{max}-r_{l}}\right)$, $X_{2}=t-t_{max}$. Then, non-linear Equations (6) and (7) are converted into the linear Equations (8) and (9), respectively.

(8) $$Y_{1}=-\frac{1}{\tau_{a}}X_{1}$$

(9) $$Y_{2}=-\frac{1}{\tau_{r}}X_{2}$$

**Figure 16:**
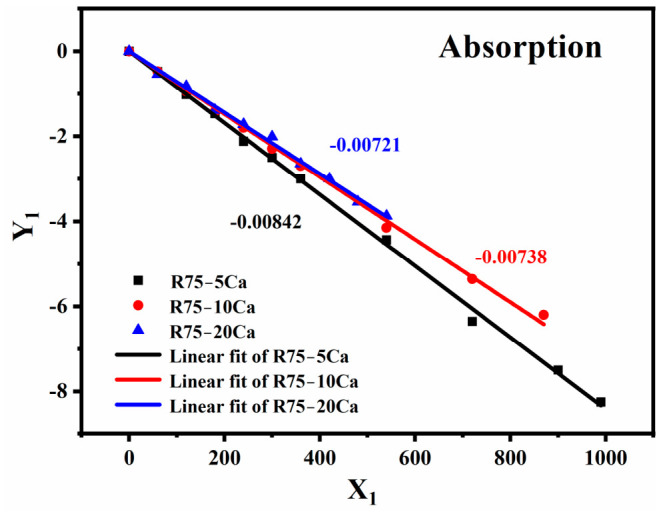
The fitting results for the absorption curves of R75-5Ca, R75-10Ca and R75-20Ca, using Equation (8) (SAP size: radius of 75 ± 20 μm; Solutions: 5 mM, 10 mM and 20 mM Ca^2+^, respectively).

**Figure 17:**
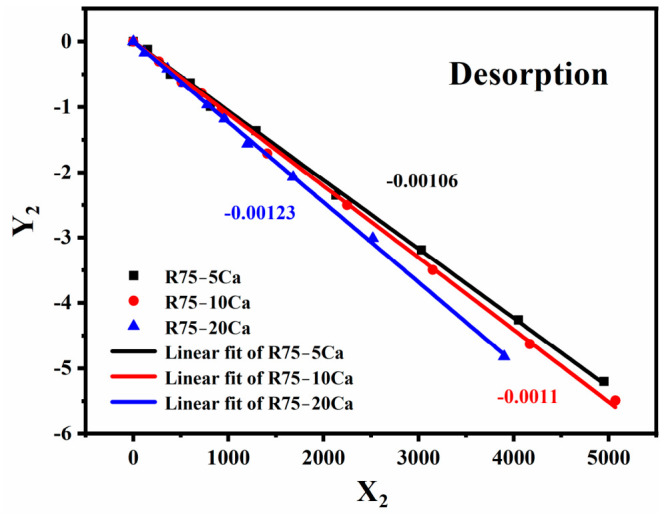
The fitting results of the desorption curves of R75-5Ca, R75-10Ca and R75-20Ca, using Equation (9) (SAP size: radius of 75 ± 20 μm; Solutions: 5 mM, 10 mM and 20 mM Ca, respectively).

The ab- and desorption curves of groups R75-5Ca, R75-10Ca and R75-20Ca are used as examples to show the fitting effects (see Figure 16 and Figure 17).

Table 2 shows the characteristic time constants $\tau_{a}$ and $\tau_{r}$ for each experimental group (Table 1), obtained by fitting the available ab- and desorption data point (Figure 13, Figure 14 and Figure 15) using Equations (8) and (9). In addition, the typical ab- and desorption curves from the literature [26] (marked as R300-24Ca-L) were also utilized for fitting. The fitted values of $\tau_{a}$ and $\tau_{r}$ are listed in Table 2 as well. The fitting effects are positive in terms of the degree of fitting ($R^{2}$). In other words, the boundary conditions of the deformation equations can be well expressed by Equations (6) and (7).

### 4.6. Model Development

The model couples two primary physical processes: ion transport within the SAP and its subsequent elastic deformation.

#### 4.6.1. Ion Transport Model of SAP

In the context of ion exchange between monovalent ions (A^+^, e.g., Na^+^) and higher valent ions (B^z+^, e.g., Ca^2+^), the diffusional characteristics of the exchanged ions within the resin phase exhibit a non-stationary nature. This implies that the temporal and spatial distribution of ion concentration in the resin phase varies dynamically. Non-stationary diffusion processes of this nature are commonly addressed through the utilization of Fick’s second law. Specifically, for the case of spherical SAP described in spherical coordinates, Fick’s second law can be mathematically expressed as follows.

(10) $$\frac{\partial C_{all}}{\partial t}=\frac{-1}{r^{2}}\frac{\partial }{\partial r}\left(r^{2}\cdot J\right)$$

For exchange ion B:

(11) $${C_{all}=C}_{RB}+C_{B}$$

where $C_{all}$ is the total concentration of exchange ion B in resin phase (SAP), $C_{B}$ is the concentration of exchange ion B dissociated in SAP and $C_{RB}$ is the concentration of exchange ion B solidified in SAP. In the actual transport process, only the dissociated exchange ion B is involved in the diffusion process and the diffusion flux $J_{B}$ is expressed in Equation (12).

(12) $$J_{B}=-D_{AB}\frac{\partial C_{B}}{\partial r}$$

where $D_{AB}$ is the mutual diffusion coefficient. By bringing Equations (11) and (12) into Equation (10), the above equation can be written as Equation (13).

(13) $$\frac{\partial \left(C_{RB}+C_{B}\right)}{\partial t}=\frac{1}{r^{2}}\frac{\partial }{\partial r}\left(r^{2}\cdot D_{AB}\frac{\partial C_{B}}{\partial r}\right)\;\;$$

The degree of dissociation (k) of the counter ion is expressed in Equation (14).

(14) $$\frac{C_{B}}{C_{B}+C_{RB}}=k$$

The dissociation degree k is treated as a constant in the present model, which is a simplification. In reality, k depends on local Ca^2+^ and Na^+^ concentrations, pH, carboxylate availability, and crosslink density. For weak acid resins (e.g., polyacrylate), however, experimental measurements have shown that k for Ca^2+^ varies within a relatively narrow range (0.002–0.008) under conditions similar to ours. We therefore use a constant k as an approximation. For example, when B is the Ca^2+^ ion, the equilibrium equation for the dissociation of Ca^2+^ in resin is expressed as follows.

(15) $$(R-COO^{-}{)}_{2}{Ca}^{2+}⇌{Ca}^{2+}+2R-COO^{-}$$

where $(R-COO^{-}{)}_{2}{Ca}^{2+}$ is the crosslinked Ca^2+^ ions in resin phase, Ca^2+^ is the free Ca^2+^ concentration in resin phase and $R-COO^{-}$ is the carboxyl group. The dissociation degree of Ca^2+^ is expressed in Equation (16).

(16) $$\frac{C_{Ca}}{C_{Ca}+C_{R_{2}Ca}}=k$$

$D_{AB}$ is related to the ion concentration in the exchange process [39], which makes solving the partial differential equation quite difficult. Therefore $D_{AB}$ is usually replaced by the harmonic average of the self-diffusion coefficients of the A and B ions in the resin phase (marked as $D_{r}$), as shown in Equation (17):

(17) $$D_{r}=\frac{D_{A}D_{B}(Z_{A}^{2}+Z_{B}^{2})}{Z_{A}^{2}D_{A}+Z_{B}^{2}D_{B}}\;\;$$

where $D_{A}$ and $D_{B}$ are the self-diffusion coefficient of the A and B ions in the resin phase, respectively, and $Z_{A}$ and $Z_{B}$ are the charge numbers of the A and B ions, respectively. $D_{A}$ and $D_{B}$ are intensely affected by porosity (∅) of SAP [40,41], as is expressed in Equations (18) and (19).

(18) $$D_{A}=D_{fA}{\left(\frac{2\emptyset }{3-\emptyset }\right)}^{2}$$

(19) $$D_{B}=D_{fB}{\left(\frac{2\emptyset }{3-\emptyset }\right)}^{2}$$

where $D_{fA}$ and $D_{fB}$ are the self-diffusion coefficients of the A and B ions in water, respectively, and ∅ is the porosity of SAP. The final equation for the ion diffusion transport in spherical SAP is expressed as Equation (20).

(20) $$\frac{\partial C_{B}}{\partial t}=\frac{1}{r^{2}}\frac{\partial }{\partial r}\left[kr^{2}{\cdot D}_{r}\frac{\partial C_{B}}{\partial r}\right]\;\;$$

Clearly, $D_{r}$ is not a constant, but a time- and space-dependent variable, and the equation has only numerical solutions.

The model is developed for polyacrylate-type SAPs with carboxylate functional groups, which are the SAPs most widely used in construction and hygiene products. The ion-exchange reaction with Ca^2+^ is written as Equation (6). For other SAP chemistries (e.g., sulfonate-based gels), the binding stoichiometry and dissociation degree k would need recalibration.

#### 4.6.2. Deformation Model of SAP

**Figure 18:**
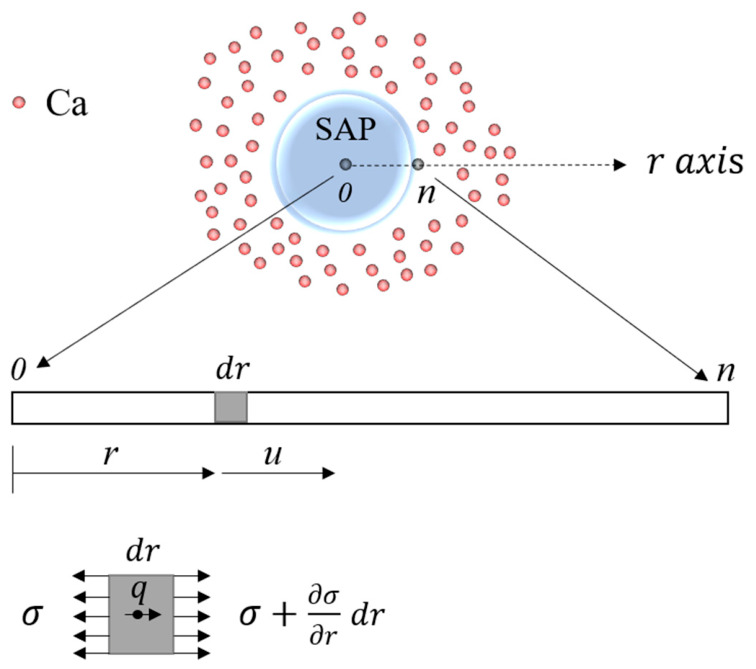
Schematic map of force analysis for SAP deformation. The SAP in the r-axis direction is regarded as a thin rod.

The swelling of SAP (hydrogels) can be considered as elastic deformation despite its large volume strain (almost up to 50,000%) [42]. Therefore, the basic elastic theory (kinetic equations) is still well suited for application to the SAP deformation. For the sake of simplicity, the deformation dynamics equation of SAP was derived in a rectangular coordinate system using the 1D rod model (with section area A, Young’s modulus E and density ρ) shown in Figure 18 as an example. Finally, we will unify it into polar coordinates. The force analysis for the unit body in the r-axis (horizontal direction) is shown in Figure 2.

In Figure 18, u is the horizontal displacement at each point during deformation of SAP; u(r,0)=0, q is the body stress of the unit body; and σ is the axial stress. Based on Newton’s second law,

(21) $$-\sigma A+\left(\sigma +\frac{\partial \sigma }{\partial r}dr\right)A+qAdr=\rho Adr\frac{\partial^{2}u}{\partial t^{2}}\;\;\;$$

Namely,

(22) $$\frac{\partial \sigma }{\partial r}+q=\rho \frac{\partial^{2}u}{\partial t^{2}}\;$$

Based on the generalized Hooke’s law σ=Eε and the strain equation $\varepsilon =\frac{\partial u}{\partial r}$, Equation (22) can be written as Equation (23):

(23) $$\frac{\partial }{\partial r}\left(E\frac{\partial u}{\partial r}\right)+q=\rho \frac{\partial^{2}u}{\partial t^{2}}$$

To simplify the calculation, the volumetric stress *q* is omitted. The unit body has similar relationships with the other two pairs of parallel surfaces. For three dimensions, the expression is Equation (24), where ∇ is the gradient operator:

(24) $$\rho \frac{\partial^{2}u}{\partial t^{2}}=\nabla \cdot \left(E\nabla u\right)$$

In spherical coordinates (for spherical SAP), the fluctuation equation is expressed using Equation (25).

(25) $$\rho \frac{\partial^{2}u}{\partial t^{2}}=\frac{1}{r^{2}}\frac{\partial }{\partial r}\left[r^{2}\cdot E\frac{\partial u}{\partial r}\right]\;$$

In this study, the kinetic equations governing the absorption and desorption processes of superabsorbent polymers (SAPs) in solution are derived by incorporating the principles of elasticity theory. Specifically, Equation (25), presented in this work, corresponds to the widely recognized standard spherical wave equation [43].

It should be noted that the elastic wave equation employed here serves as a first-order phenomenological approximation for capturing the dynamic deformation of SAPs under spherical symmetry. Although the volume strain can be as high as 50,000%, the model does not purport to be a complete finite-strain or poroelastic hydrogel swelling theory. Rather, it provides a simplified mechanical framework that, when coupled with ion transport, can reproduce the experimentally observed swelling/deswelling kinetics and predict emergent chemo-mechanical behaviors. Future work should incorporate finite-strain elasticity and osmotic pressure explicitly.

#### 4.6.3. Coupling Mechanism

The mathematical models of ion transport and SAP deformation have been given in Section 4.6.1 and Section 4.6.2, respectively. In fact, the two models are closely related and interactive. This section deals with the coupling effect of SAP deformation and ion transport, which is also the focus in our models.

(a) The effect of ion transport on SAP deformation: One important fact is that the Young’s modulus of the SAP is not only influenced by the porosity of the SAP [44], but also by the concentration of cross-linked B ions [27]. In our model, the Young’s modulus is expressed using Equation (26), which is composed of two terms. The first term is the contribution of porosity to the modulus, following the classical elasto-plastic theory. The second term is the contribution of cross-linked B ions to the modulus. Although the relationship between modulus and the concentration of cross-linked B ions is not given in the works in the literature, luckily, some quantitative data are provided [27,44]. Through integrating and analyzing the data from the literature, we make a linear assumption between them (see Equation (26)).

(26) $$E=E_{0}{\left(1-\emptyset \right)}^{1/3}+wC_{RB}$$

where w is the linear coefficient and $C_{RB}$ is the concentration of cross-linked B ions to the carboxyl group. w was estimated by fitting Equation (17) to the limited experimental data from Refs. [27,44], where the elastic modulus of polyacrylate SAPs was reported as a function of Ca^2+^ concentration. The linear coefficient was obtained by regression over the $C_{RB}$ range of 0 to approximately 50 mM covered in those studies [27,44]. The linear relationship between the elastic modulus E and the concentration of crosslinked B ions $C_{RB}$ is adopted as a first approximation, following the observation that modulus increases with ionic crosslink density in polyacrylate hydrogels. However, we acknowledge that ionic crosslinking can exhibit nonlinear, cooperative, or saturation behavior. To assess the robustness of our model predictions, we performed a sensitivity analysis using alternative nonlinear forms (e.g., $E=E_{0}{\left(1-\emptyset \right)}^{1/3}+w{C_{RB}}^{n}$ with *n* < 1). The qualitative phenomena (concentration gradient inversion and free Ca^2+^ overshoot) persist for a range of nonlinear exponents (see Figure S7 in the Supplementary Materials), suggesting that the linear assumption does not introduce artificial artifacts. A direct experimental measurement of modulus evolution as a function of our tested Ca^2+^ concentrations is planned for future work.

It is clear from Equation (26) that E is not a constant, but a time- and space-dependent variable. Therefore, similarly to the transport equation Equation (20), Equation (25) is also a variable coefficient partial differential equation and can only be solved numerically. It needs to be pointed out that in our models, the water transport is regarded as an instantaneous process. In other words, the effect of water transport on E of the SAP is uniform.

(b) The effect of SAP deformation on Ca^2+^ ion transport is reflected in two aspects: (1) As to the effect of SAP deformation on the diffusion coefficient ($D_{r}$), the deformation (referring to water ab- and desorption of the SAP) inevitably generates a volume strain (θ), which also changes the porosity of the SAP (∅). The relationship between ∅ and θ is shown in Equation (27). And the relationship between θ and displacement (u) is shown in Equation (28). Based on Equations (17)–(19), $D_{r}$ is a function of ∅; therefore, $D_{r}$ is also a function of u. In other words, the deformation of a SAP will inevitably change the diffusion coefficient ($D_{r}$). (2) As to the effect of SAP deformation on free B ion concentration ($C_{B}$), similarly, θ leads to the change of $C_{B}$. It is important to note that the volume strain used to calculate $C_{B}$ at moment t+dt is the relative volume strain (marked as $\theta^{*}$) at adjacent moments t and t+dt, not that (θ) at moments 0 and t+dt. According to this definition, $\theta^{*}$ is related to u as shown in Equation (29). And the relationship between $C_{B}$ and $\theta^{*}$ is shown in Equation (30).

(27) $$\emptyset \left(r,t\right)=1-\frac{1-\emptyset_{0}}{\theta \left(r,t\right)+1}\;$$

(28) $$\left(r,t\right)=\frac{V\left(r,t\right)-V\left(r,0\right)}{V\left(r,0\right)}{=\left(1+\frac{u\left(r,t\right)}{r}\right)}^{2}\left(1+\frac{u\left(r+dr,t\right)-u\left(r,t\right)}{dr}\right)-1$$

where $\emptyset_{0}$ is the porosity of dry SAP.

(29) $$\begin{array}{cc} \theta^{*}\left(r,t+dt\right)= & \frac{V\left(r,t+dt\right)-V\left(r,t\right)}{V\left(r,t\right)}\\ & =\frac{{\left(r+u\left(r,t+dt\right)\right)}^{2}(dr+u\left(r+dr,t+dt\right)-u(r,t+dt))}{{\left(r+u\left(r,t\right)\right)}^{2}(dr+u\left(r+dr,t\right)-u(r,t))} \end{array}$$

(30) $$C_{B}\left(r,t+dt\right)=\frac{C_{B}\left(r,t\right)}{1+\theta^{*}}$$

**Figure 19:**
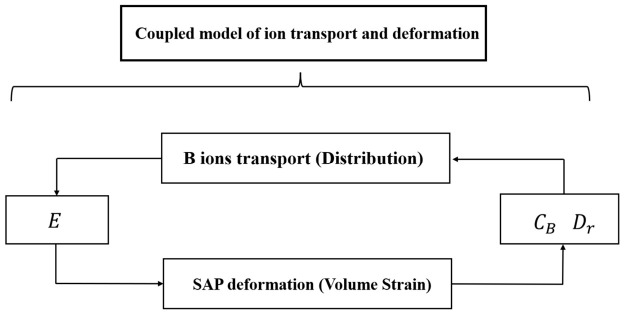
The roadmap of the coupled model of the transport of B ions and SAP deformation.

In summary, the internal logic of the whole model can be expressed using the roadmap in Figure 19.

#### 4.6.4. Numerical Solution

As mentioned above, both the ions transport equation and the deformation equation are partial differential equations with variable coefficients, and it is quite difficult to obtain their analytical solutions. Therefore, in this paper, both of the equations were numerically solved using the finite difference method [33,45]:

As for the ions transport equation Equation (31), it can be derived and combined into Equation (32).

(31) $$\frac{\partial C}{\partial t}=\frac{\partial }{\partial r}(kD_{r}\frac{\partial C}{\partial r})+\frac{2kD_{r}}{r}\frac{\partial C}{\partial r}$$

The formula expands to

(32) $$\frac{\partial C}{\partial t}=\frac{\partial (kD_{r})}{\partial r}\frac{\partial C}{\partial r}+kD_{r}\frac{\partial^{2}C}{\partial r^{2}}+\frac{2kD_{r}}{r}\frac{\partial C}{\partial r}$$

Let $D=D_{r},\alpha =\frac{D_{r}}{r}$. Write Equation (32) in differential format as

(33) $$\begin{array}{cc} \frac{1}{\tau }\left(C_{j}^{k}-C_{j}^{k-1}\right)= & \frac{1}{2}kD_{j}^{k-1}\frac{C_{j+1}^{k}-2C_{j}^{k}+C_{j-1}^{k}}{h^{2}}+k\frac{D_{j+1}^{k-1}-D_{j-1}^{k-1}}{2h}\frac{C_{j+1}^{k}-C_{j-1}^{k}}{2h}\\ & +2k\frac{{\alpha_{j+1}^{k}C}_{j+1}^{k}-\alpha_{j}^{k}C_{j-1}^{k}}{2h} \end{array}$$

where h is the spatial step, τ is the time step, and $C_{j}^{k}$ is the concentration of B ions at space j and time k. As for the deformation equation, Equation (25), it is combined after derivation as

(34) $$\frac{\partial^{2}u}{\partial t^{2}}=\frac{\partial }{\partial r}(E\frac{\partial u}{\partial r})+\frac{2E}{r}\frac{\partial u}{\partial r}$$

Let $\beta =\frac{2E}{r}$. Write Equation (34) in differential format as

(35) $$\begin{array}{l} \frac{1}{\tau^{2}}\left(u_{j}^{k+1}-{2u}_{j}^{k}+u_{j}^{k-1}\right)=\frac{g}{h^{2}}\left[E_{j+1}^{k}\left(u_{j+1}^{k+1}-u_{j}^{k+1}\right)-E_{j}^{k}\left(u_{j}^{k+1}-u_{j-1}^{k+1}\right)\right]\\ +\frac{\left(1-2g\right)}{h^{2}}\left[E_{j+1}^{k}\left(u_{j+1}^{k}-u_{j}^{k}\right)-E_{j}^{k}\left(u_{j}^{k}-u_{j-1}^{k}\right)\right]\\ +\frac{g}{h^{2}}\left[E_{j+1}^{k}\left(u_{j+1}^{k-1}-u_{j}^{k-1}\right)-E_{j}^{k}\left(u_{j}^{k-1}-u_{j-1}^{k-1}\right)\right]+\frac{{\beta_{j+1}^{k}u}_{j+1}^{k}-{\beta_{j}^{k}u}_{j-1}^{k}}{2h} \end{array}$$

where g  is the difference coefficient, and in this paper g = 0.5; $u_{j}^{k}$ is the displacement of unit body at space j and time *k*; and $E_{j}^{k}$ is the elastic modulus of unit body at space j and time k.

Before solving Equations (20) and (25) numerically, it is necessary to clarify the initial conditions and boundary conditions. For Equation (20), the initial condition is $C_{B}\left(r,0\right)=0$; the boundary condition is $C_{B}\left(r_{0},0\right)=C_{0}$, where $r_{0}$ is the outermost layer of the spherical SAP; and $C_{0}$ is the concentration of B ion in the solution. For Equation (25), the initial condition is u(r,0)=0, and the boundary condition is the formula describing the ab- and desorption curves of the SAP, which is discussed in Section 4.2 in detail. The boundary condition $C_{B}\left(r_{0},0\right)=C_{0}$ (constant surface concentration) was applied under the assumption that the external solution volume is sufficiently large to maintain a constant bulk Ca^2+^ concentration. A mass-balance calculation confirmed that for a 500 mL solution containing 0.3 g of dry SAP, the maximum relative change in bulk Ca^2+^ concentration is less than 1.2% over the entire experiment. For smaller solution volumes or higher SAP mass fractions, a time-dependent boundary condition would be necessary; this is noted as a consideration for future extensions of the model.

In addition, some important initial calculation parameters are summarized as follows. The porosity, density and elastic modulus of dry SAP are 0.59, 0.685 g/cm^3^ and 59.25 N/m^2^, respectively, which is consistent with the parameters in the literature [34]. And the diffusion coefficients of the Ca^2+^ and Na^+^ ions in water are 1.33 × 10^−9^ m^2^/s and 0.79 × 10^−9^ m^2^/s, respectively.

#### 4.6.5. Numerical Implementation Details

To ensure reproducibility [32], we provide the following details of the finite-difference scheme:

- Spatial discretization: The spherical SAP radius is divided into *N* = 200 equally spaced nodes with spacing *h* = *r*_0_/*N*.
- Time discretization: Constant time step *τ* = 0.001 s.
- Boundary conditions: At *r* = 0, symmetry gives $\frac{{\partial C}_{B}}{\partial r}$ = 0 and *u* = 0. At *r* = *r*_0_(*t*), $C_{B}$ = $C_{0}$ and *u* follows the fitted radius–time curve (Equations (4) and (5)).
- Solution procedure: The ion diffusion equation (Equation (20)) is solved using the Crank–Nicolson implicit scheme to avoid the divergence issue. The deformation equation (Equation (25)) is solved using the explicit central difference scheme with g = 0.5, which corresponds to a leapfrog method. Stability is ensured by τ ≤ *h*/$\sqrt{E/\rho }$.

As the time step and spatial step do not significantly influence the gradient reversal results (see Figures S8 and S9), the above parameters were maintained unchanged throughout the present study.

## 5. Limitations and Future Work

The present model contains several simplifications that should be considered when interpreting its results.

Mechanical formulation: The elastic wave equation with small-strain kinematics is a phenomenological approximation to the true finite-strain viscoporoelastic swelling of hydrogels. It does not resolve osmotic pressure, solvent chemical potential, or polymer-network entropy [7,46]. The model is thus best viewed as a tool for exploring the first-order coupling between deformation and ion transport, rather than a complete constitutive theory.

Water transport assumption: The assumption of instantaneous water transport, i.e., uniform water distribution at any time, is a deliberate simplification to isolate the chemo-mechanical coupling between ion transport and deformation. In deionized water, this assumption is supported by the experimental observation of a nearly moisture-gradient-free swelling process. In Ca^2+^-containing solutions, however, local desorption and crosslinking may introduce spatial gradients in water content. We caution that the instantaneous water transport assumption may become limiting for very large SAP particles or highly crosslinked systems [37]. A model output considering water transport is shown in Figure S10 in the Supplementary Materials. Determination of a full poroelastic formulation remains an important direction for future research.

Ion–modulus coupling: The linear relationship $E=E_{0}{\left(1-\emptyset \right)}^{1/3}+wC_{RB}\;$ is a first-order approximation validated only over a limited $C_{RB}$ range. Although our sensitivity analysis (see the Supplementary Materials) shows that the key phenomena are robust to the functional form, direct experimental measurement of the E-$C_{RB}$ relationship under controlled Ca^2+^ concentrations is needed.

Dissociation degree: k is treated as a constant, whereas in reality it depends on local ion concentrations, pH, ionic strength, and crosslink density. Developing a local-equilibrium binding isotherm would improve the model’s generality.

Experimental validation of internal fields: The predicted internal gradients of free Ca^2+^ concentration, elastic modulus, and volumetric strain have not been directly measured. Future work should employ confocal laser scanning microscopy with Ca^2+^-sensitive fluorescent indicators (e.g., Fluo-4), micro-CT imaging, or Raman/FTIR mapping to validate these spatial distributions [47].

History and environmental effects: The model does not account for swelling/deswelling history, network aging, or mechanical confinement. Extensions to incorporate these effects, possibly via time-dependent crosslink density evolution and external mechanical boundary conditions, would be valuable for applications such as SAP in hydrating cement paste. The framework can be extended to incorporate external mechanical constraints in two ways: (1) modifying the deformation boundary condition at the SAP surface (e.g., prescribing a confining pressure or linking it to a poroelastic model of the surrounding medium); or (2) adding an external stress term to the governing wave equation (Equation (25)).

Real cement pore solutions: The real cement pore solutions contain high pH, alkalis, sulfate, aluminates, silicates, and evolving ionic strength [48]. Calcium behavior in cement pore fluid is not equivalent to that in a simple Ca^2+^ solution. Our simplified system is a first step, and future work should include synthetic pore solutions or real cement extracts.

## Figures and Tables

**Table 1:** The detailed information for the experimental groups. $C_{Ca}$ and $C_{Na}$ represent the concentrations of Ca^2+^ and Na^+^ cations, respectively.

Experiment Groups	SAP Size (μm)	$C_{Ca}$ (mmol/L)	$C_{Na}$ (mmol/L)
R75-0Ca	75	0	0
R200-0Ca	200	0	0
R300-0Ca	300	0	0
R75-20Ca	75	20	360
R200-20Ca	200	20	360
R300-20Ca	300	20	360
R75-5Ca	75	5	360
R75-10Ca	75	10	360
R300-24Ca-L	300	24	0

**Table 2:** The characteristic time constants of the absorption curves ($\tau_{a}$ in Equation (6)) and desorption curves ($\tau_{r}$ in Equation (7)) for each group in Table 1, as obtained by fitting.

Experiment Group	$\tau_{a}$ (s)	$\tau_{r}$ (s)	Degree of Fitting $R^{2}$
R75-0Ca	98.32	/	0.9911
R200-0Ca	107.53	/	0.9930
R300-0Ca	111.12	/	0.9920
R75-20Ca	138.70	813.00	0.9960/0.9936
R200-20Ca	158.73	909.23	0.9937/0.9927
R300-20Ca	178.57	939.15	0.9927/0.9935
R75-5Ca	118.76	943.40	0.9944/0.9954
R75-10Ca	135.50	909.09	0.9935/0.9945
R300-24Ca-L	71.23	3333.34	0.9941/0.9569

## Data Availability

The raw data supporting the conclusions of this article will be made available by the authors on request.

## Supplementary Materials

The following supporting information can be downloaded at: https://www.mdpi.com/article/10.3390/gels12070606/s1, Figure S1: The spatial and temporal distribution of elastic modulus of spherical SAP with three dry particle sizes (with radius of 75 μm, 200 μm and 300 μm) in solutions containing 20 mM Ca^2+^: (a–c) Spatial distribution; (d–f) temporal distribution. Figure S2: The spatial and temporal distribution of elastic modulus of spherical SAP (with a radius of 75 μm) in solutions containing three kinds of Ca^2+^ concentration (5 mM, 10 mM and 20 mM): (a–c) Spatial distribution; (d–f) temporal distribution. Figure S3: SAP particle size distribution of 75 ± 20 μm. Figure S4: Relationship between SAP diameter and mass. Figure S5: Conversion relationship between SAP volume and mass. Figure S6: Effect of SAP particle size on the concentration reversal of free Ca ions. Figure S7: A sensitivity analysis using nonlinear modulus-concentration relationships, where n is the exponent of the concentration $C_{RB}$ in the formula ($E=E_{0}{\left(1-\emptyset \right)}^{1/3}+w{C_{RB}}^{n}$). Figure S8: Effect of time step on the distribution of free Ca ions. Figure S9: Effect of spatial step on the distribution of free Ca ions. Figure S10: Distribution of free Ca ions within SAP with and without considering water transport.

## Nomenclature

Symbol	Definition	Unit
(a) Ion transport		
*C_all_*	Total concentration of exchange ion B in resin phase (SAP)	mol/m^3^
*C_B_*	Concentration of dissociated (free) ion B in SAP	mol/m^3^
*C_RB_*	Concentration of crosslinked (bound) ion B in SAP	mol/m^3^
*C_Ca_*	Free Ca^2+^ concentration in the SAP	mol/m^3^
*C_R_*_2_ *_Ca_*	Crosslinked Ca^2+^ concentration in the SAP	mol/m^3^
*C*_0_	Concentration of ion B in external solution	mol/m^3^
*C*_0_ *_r_*	Concentration of ion B at the outermost boundary of the SAP	mol/m^3^
*J*	Diffusion flux	mol/(m^2^·s)
*J_B_*	Diffusion flux of ion B	mol/(m^2^·s)
*D_AB_*	Mutual diffusion coefficient	m^2^/s
*D_r_*	Harmonic average self-diffusion coefficient of ions A and B in resin phase	m^2^/s
*D_A_*	Self-diffusion coefficient of ion A in resin phase	m^2^/s
*D_B_*	Self-diffusion coefficient of ion B in resin phase	m^2^/s
*D_fA_*	Self-diffusion coefficient of ion A in free water	m^2^/s
*D_fB_*	Self-diffusion coefficient of ion B in free water	m^2^/s
*k*	Degree of dissociation of counterion	–
*Z_A_*	Charge number of ion A	–
*Z_B_*	Charge number of ion B	–
A^+^	Monovalent counterion (e.g., Na^+^)	–
B^2+^	Divalent counterion (e.g., Ca^2+^)	–
(b) Deformation and mechanics		
*u*	Radial displacement	m
*r*	Radial coordinate	m
*r*_0_	Initial (dry) radius of the SAP	μm
*r_max_*	Maximum radius of the SAP (at peak absorption)	μm
*r_l_*	Final radius of the SAP	μm
*r*(*t*)	Radius of the SAP at time t	μm
*r_i_*	Radial position of the i-th spatial node	m
*σ*	Axial stress	Pa
*ε*	Strain	–
*θ*	Volumetric strain	–
*θ* ^*^	Relative volumetric strain between adjacent time steps t and t + dt	–
*E*	Young’s modulus	Pa
*E*_0_	Initial Young’s modulus of dry SAP	Pa
*w*	Linear coefficient for modulus contribution of crosslinked ion B	Pa·m^3^/mol
*q*	Body force per unit volume	N/m^3^
*ρ*	Density of SAP	kg/m^3^
*Φ*	Porosity of SAP	–
*Φ*_0_	Initial porosity of dry SAP	–
(c) Time and kinetic parameters		
*t*	Time	s or min
*t_max_*	Time at which the SAP reaches maximum radius (maximum absorbency)	s
*τ*	Tanaka–Fillmore characteristic time constant	s
*τ_a_*	Characteristic time constant for absorption	s
*τ_r_*	Characteristic time constant for desorption	s
*D_coop_*	Cooperative diffusion coefficient	m^2^/s
(d) Numerical solution		
*h*	Spatial step size	m
Δ*t*	Time step size	s
*C_j_^k^*	Concentration of ion B at spatial node j and time point k	mol/m^3^
*u_j_^k^*	Displacement at spatial node j and time point k	m
*E_j_^k^*	Elastic modulus at spatial node j and time point k	Pa
*D_j_^k^*	Diffusion coefficient at spatial node j and time point k	m^2^/s
*g*	Difference coefficient (taken to be 0.5 in this study)	–
*α*	Abbreviation for D_r_/r in numerical scheme	m/s
*β*	Abbreviation for 2E/r in numerical scheme	N/m^3^
*i*	Spatial node index	–
*j*	Spatial node index	–
*M*	Total number of spatial nodes	–
(e) Experimental and validation		
*A_t_*	Absorption capacity of SAP in liquid at time t	g/g
*A*(*t*)	Total amount of Ca^2+^ trapped by a single spherical SAP at time t	mol
*m*_1_	Exact mass of dry SAP	g
*m*_2_	Mass of tea bag	g
*m*_3_	Total mass of tea bag and swollen SAP	g
*V*(*r*, *t*)	Volume of SAP at position r and time t	m^3^
*R* ^2^	Coefficient of determination (goodness of fit)	–
