# Supplementary material for: A Phenomenological Coupled Model for Ion Transport and Deformation in Superabsorbent Polymers in Calcium-Containing Solutions
Source: Gels. 2026 Jul 7;12(7):606. doi: 10.3390/gels12070606 (PMC13409075; doi:10.3390/gels12070606)
Supplement: Supplementary file 1 [file gels-12-00606-s001.zip › gels-4312896-supplementary.pdf]

Supplementary materials

(a) Effect of SAP size on elastic modulus in solution containing 20 mM Ca^{2+}

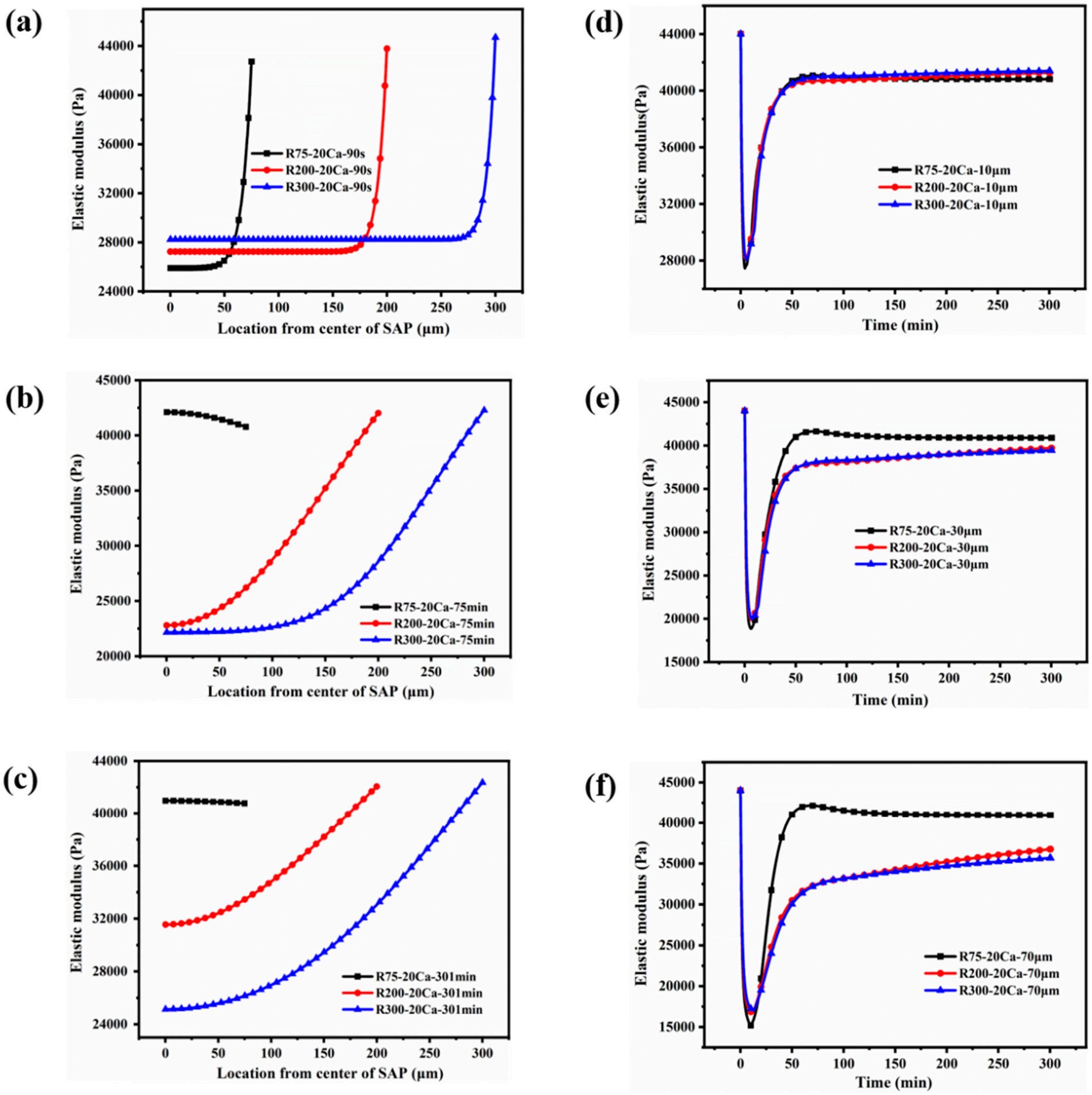

Figure S1. The spatial and temporal distribution of elastic modulus of spherical SAP with three dry particle sizes (with radius of 75 μm, 200 μm and 300 μm) in solutions containing 20 mM Ca^{2+}: (a)-(c) Spatial distribution; (d)-(f) temporal distribution.

(b) Effect of Ca^{2+} concentration on elastic modulus

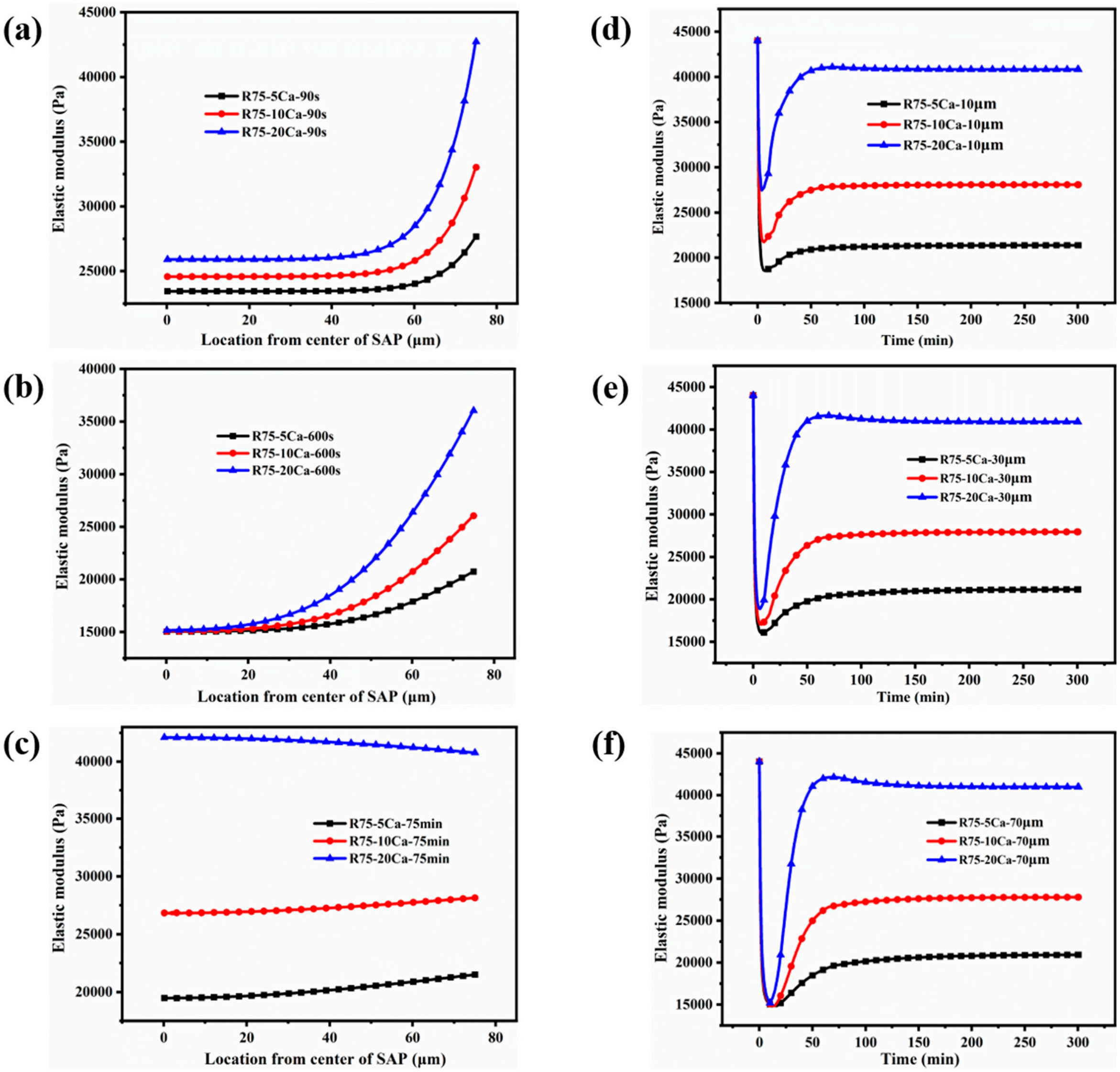

Figure S2. The spatial and temporal distribution of elastic modulus of spherical SAP (with a radius of 75 μm) in solutions containing three kinds of Ca^{2+} concentration (5 mM, 10 mM and 20 mM): (a)-(c) Spatial distribution; (d)-(f) temporal distribution.

(c) Particle-size distributions and sensitivity analysis

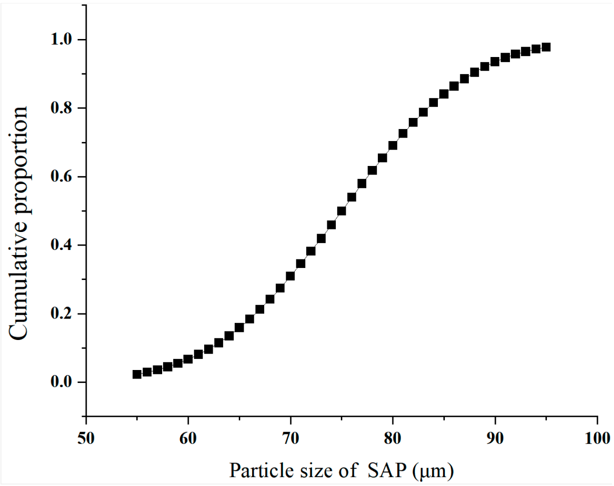

Figure S3. SAP particle size distribution of 75 ± 20 μm.

(d) Single-particle radius evolution using an average volume-to-mass relationship and provide uncertainty bounds

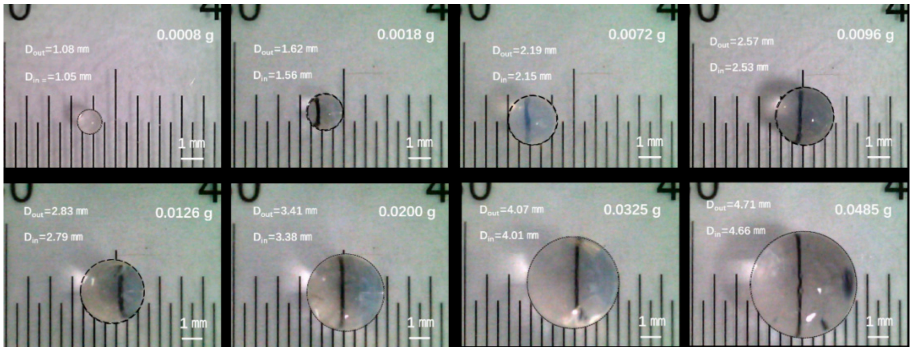

Figure S4. Relationship between SAP diameter and mass.

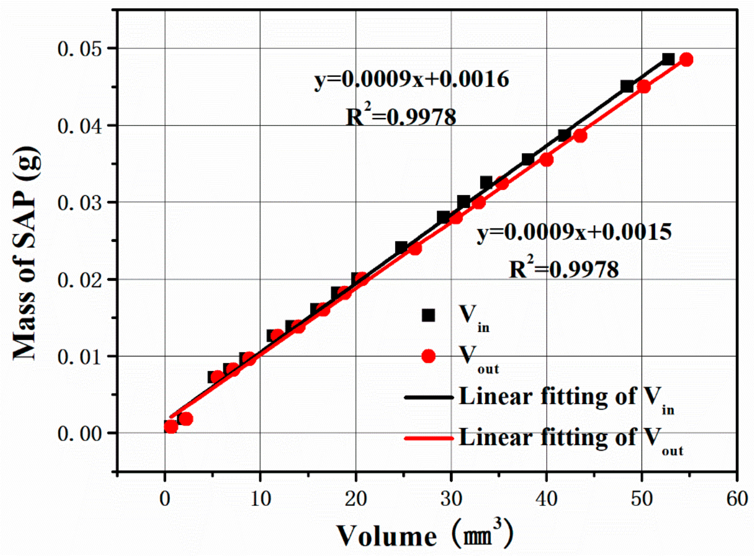

Figure S5. Conversion relationship between SAP volume and mass.

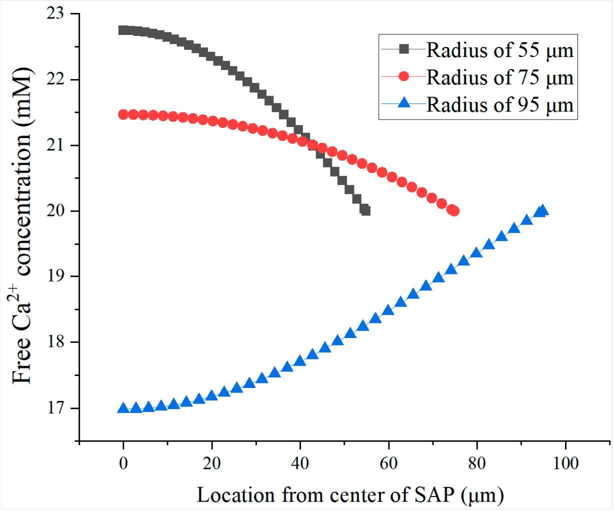

Figure S6. Effect of SAP particle size on the concentration reversal of free Ca ions.

(e) Model to describe the parameter *k*

$$\frac{\partial\left(C_{R_2Ca} + C_{Ca}\right)}{\partial t} = \frac{1}{r^2}\frac{\partial}{\partial r}\left(r^2 D_{Ca}\frac{\partial C_{Ca}}{\partial r}\right)$$

Here, C_{R_2Ca} is no longer related to C_{Ca} through a constant k, but is instead computed in real time at each spatial grid point using the aforementioned local equilibrium module. The calculation workflow is as follows.

1. Update C_{Ca} using the transport equation.
2. At each node, input C_{Ca}, C_{Na} (which can be updated based on exchange stoichiometry: for every 1 mol of Ca^{2+} bound, 2 mol of $Na^{+}$are released, so C_{Na} will also change), pH, and I (local ionic strength).
3. Solve for C_{R_2Ca}, then update the elastic modulus E (in Eq. 17, C_{RB} corresponds to C_{R_2Ca}).
4. Update the volumetric strain through the deformation equation, and iterate.

(f) A sensitivity analysis using alternative nonlinear forms(e.g., $E = E_0(1-\emptyset)^{1/3} + wC_{RB}{}^{n}$ with $n < 1$)

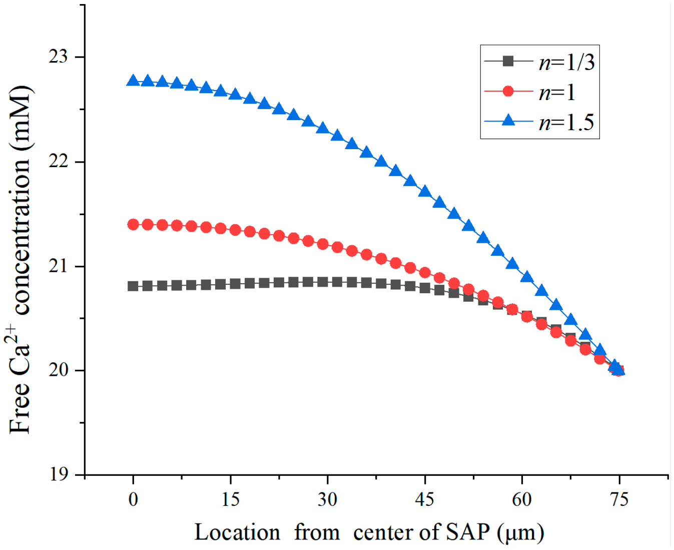

Figure S7. A sensitivity analysis using nonlinear modulus-concentration relationships, where n is the exponent of the concentration C_{RB} in the formula（$E = E_0(1-\emptyset)^{1/3} + wC_{RB}{}^{n}$）.

(g) Simulation results considering the time step、spatial step

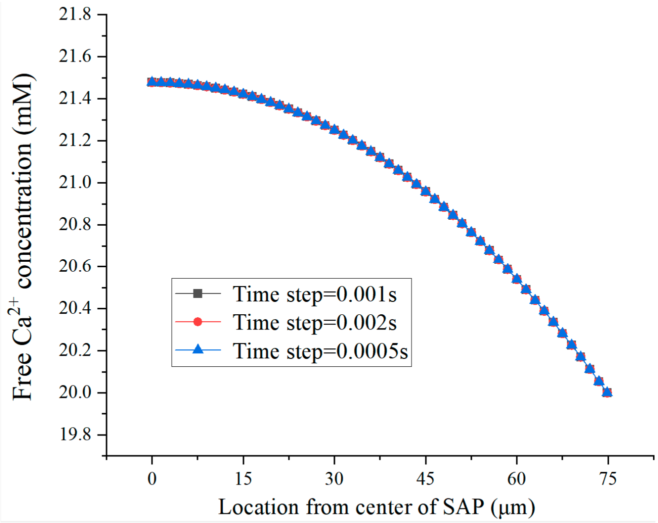

Figure S8. Effect of time step on the distribution of free Ca ions.

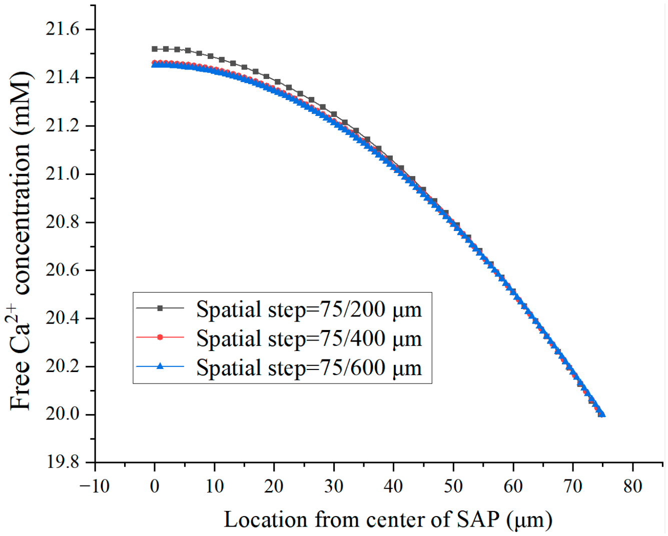

Figure S9. Effect of spatial step on the distribution of free Ca ions.

(h) Model output considering water transport

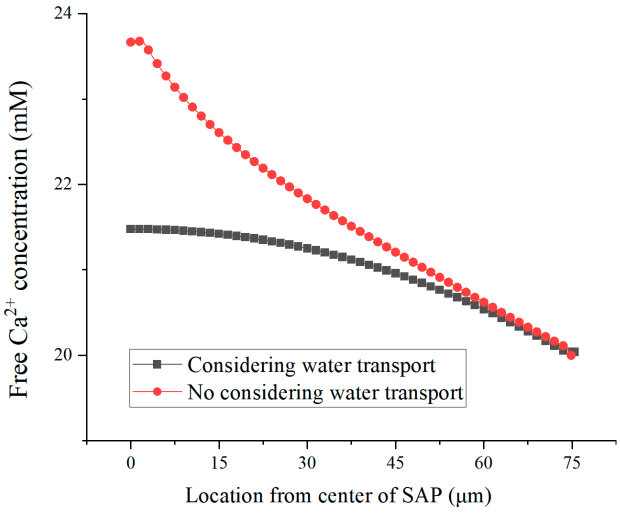

Figure S10. Distribution of free Ca ions within SAP with and without considering water transport.
